# Design of a Peripheral Building Block for H-Bonded Dendritic Frameworks and Analysis of the Void Space in the Bulk Dendrimers

**DOI:** 10.1038/s41598-017-03684-y

**Published:** 2017-06-16

**Authors:** Cheng-Hua Lee, Dmitriy V. Soldatov, Chung-Hao Tzeng, Long-Li Lai, Kuang-Lieh Lu

**Affiliations:** 1 Department of Chemistry, University of Guelph, Guelph, Ontario N1G 2W1 Canada; 2 Department of Applied Chemistry, National Chi Nan University, 1 Daxue Rd., Puli, Nantou County 545 Taiwan; 3 Institute of Chemistry, Academia Sinica, Taipei, 115 Taiwan

## Abstract

Three dendrimers, (***t***-**Bu**-**G**_**2**_ **N**)_**2**_, **CC**(***t***-**Bu**-**G**_**1**_ **N**)_**3**_ and (***t***-**Bu**-**G**_**1**_ **N**)_**2**_, with 3,5-di-*tert*-butyl amidobenzene as a common peripheral moiety were prepared in 64–83% yields and characterized. The bulk solids had high BET surface areas of 136–138 m^2^/g, which were similar for the three dendrimers in spite of their different molecular weight (ranging from 1791 to 2890). It was concluded that the peripheral amide groups do not imbed in the interstitial space of neighbouring dendrimer molecules but rather build a supramolecular architecture through strong intermolecular H-bonds. This mode of assembly generates voids in the bulk dendrimers responsible for sorption properties. The X-ray crystal structure analysis of a compound representing the peripheral moiety of the dendrimers and the FT-IR and powder-XRD data for (***t***-**Bu**-**G**_**1**_ **N**)_**2**_ suggest the proposed supramolecular structure. The isosteric heats of CO_2_ sorption (*Q*_st_) for (***t***-**Bu**-**G**_**2**_ **N**)_**2**_ were significantly higher than those for the other two dendrimers, which is consistent with the formation of a different type of voids within the interstitial space of the molecule. It is suggested that the interstitial void space can be designed and tuned to adjust its properties to a particular task, such as the separation of gases or a catalytic reaction facilitated by the dendrimer.

## Introduction

Dendrimers are tree-like, branched 3D molecules consisting of central, connecting and peripheral fragments. This complex structure makes them to exhibit versatile molecular conformations. Dendrimers have attracted a great deal of attention^1–4^ not only because of their controllable stepwise synthesis, but also because of their potential applications as catalysts^5–11^, molecular micelles^12–16^, light-harvesting molecules^17–22^, and sensors^23–28^.

**Figure 1:**
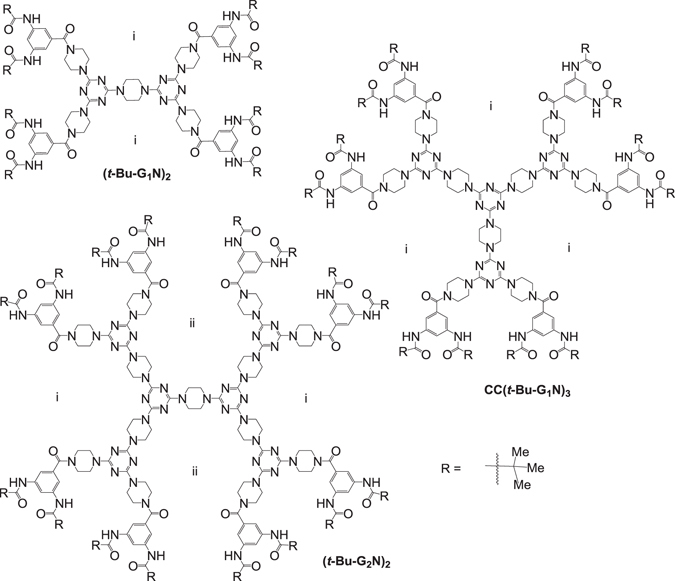
Structure of (***t*** **-Bu-G**_**2**_ **N**)_**2**_, CC(***t*** **-Bu-G**_**1**_ **N**)_**3**_, and (***t*** **-Bu-G**_**1**_ **N**)_**2**_.

On the other hand, the global warming, with the CO_2_ gas regarded as one of main causes^29–32^, calls for the development of carbon capture and sequestration technologies (CCSTs) for CO_2_ capturing and recycling^33–37^. Previously, the development of porous materials for CO_2_ adsorption focused mostly on the design of new metal–organic frameworks (MOFs)^38–42^, hydrogen-bonded organic frameworks (HOFs)^43–49^, covalent–organic frameworks (COFs)^50–55^, and polymer organic frameworks (POFs)^56–59^. MOFs and HOFs, also known as self-assembled frameworks, were studied using single crystal X-ray analysis and then the available void space could be observed at the atomic level; many of them possess high surface areas and porosity and thus show a remarkable CO_2_ sorption ability. On the other hand, COFs and POFs, consisting of carbon and other light elements, such as boron and nitrogen, in their covalent structures, generally have lower density and may show better weight percentage of adsorbed CO_2_ gas. Thus, a great deal of reports on preparation of COFs and POFs and studies of their porous properties have appeared recently. Similar to COFs and POFs, dendrimers are built of covalently bonded carbon and other light atoms and have a low density. A number of dendrimers were reported to possess molecular size void space^60–64^ adsorbing metal ions or small molecules in the interstitial space of the dendritic molecules. However, the use of dendrimers as building units in the design of porous frameworks based on intermolecular H-bond interactions has not been addressed. If the peripheral groups do not imbed in the interstitial space of the dendritic molecules in a bulk dendrimer, the void space in the supramolecular dendritic framework may be controlled as it is closely related to the interstitial space of the dendritic molecule itself (for example, the spaces i and ii in Fig. 1). Although the dendritic materials may be difficult to study by the X-ray diffraction method as in the case of POFs and COFs, their void space at the atomic level can be understood and the porosity of the overall material can be designed, as demonstrated previously for MOFs and HOFs.

The use of the triazine moiety in COFs was recently reported^65–70^. Particularly, the azaheterocyclic moiety integrated in a covalent triazine-based framework exhibits catalytic activity^68–70^. A framework made of piperazine and triazine units with a BET area of ~165 m^2^/g was found to have the CO_2_ sorption capacity as high as 5616 mg/g at 200 bar and 65 °C^65^. Previously, we reported that dendrimers containing triaminotriazine moiety show very different sorption behavior with respect to N_2_ and CO_2_ gases at high pressure; particularly, only CO_2_ and almost no N_2_ gas is adsorbed at 35 atm by the 3^rd^ generation dendrimers in the solid state^63^. This study shows that triazine-based dendrimers have a potential application in separating N_2_ from CO_2_ in a N_2_ - CO_2_ mixture. However, we were not able to identify the mode of stacking of the dendritic molecules in the solid state in our previous work^63^. With bearing all this in mind, we employed piperazine and triazine as connecting units and 3,5-di-*tert*-butylamidobenzene as a peripheral moiety in this work to prepare small dendritic molecules. We attempted to generate void space in a bulk dendritic material with the help of intermolecular H-bonds, in a way previously demonstrated for the HOFs. As there is interstitial void space in each dendritic molecule, the pore stability in the H-bonded dendritic material should not depend substantially on the encapsulated solvents.

Three dendrimers, (***t***-**Bu**-**G**_**2**_ **N**)_**2**_, **CC**(***t***-**Bu**-**G**_**1**_ **N**)_**3**_, and (***t***-**Bu**-**G**_**1**_ **N**)_**2**_, shown in Fig. 1, were prepared and studied. All three were observed to possess similar BET surface areas in spite of different molecular weights (from 1791 to 2890), which indicates that peripheral amide moieties do not embed in the interstitial space of the dendritic molecules upon their stacking in a bulk solid material. Therefore, the void space in the dendritic framework can be tuned by changing the connecting fragments of the dendrimers. In contrast to their similar sorption capacities, the isosteric heats for CO_2_ sorption (*Q*_st_) of (***t***-**Bu**-**G**_**2**_ **N**)_**2**_ are higher than those for the two others, indicating a stronger interaction and prospects for the design of materials with catalytic activity. The observed properties demonstrate the potential of triazine-based dendrimers for future applications. The details of this study are reported here.

## Results and Discussion

**Figure 2:**
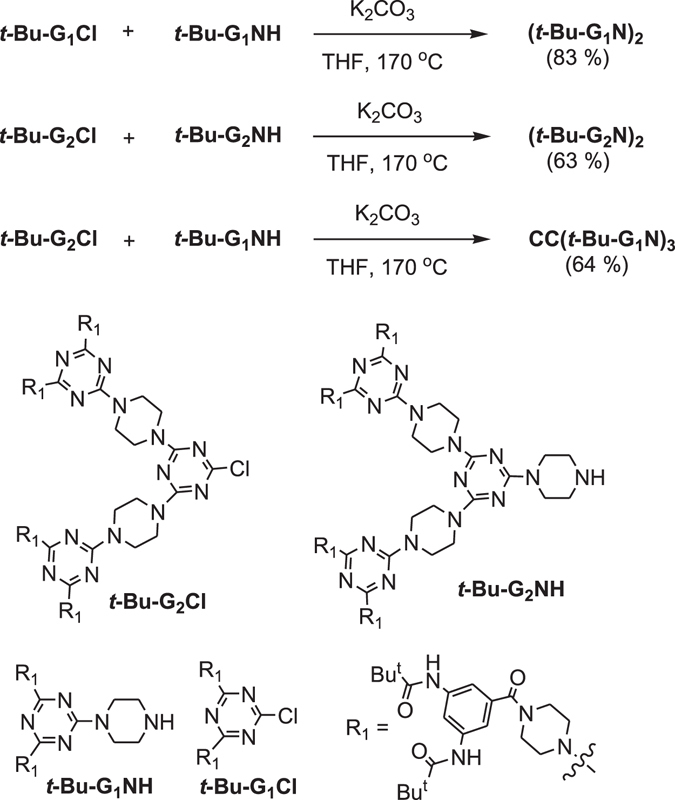
Preparation of dendrimers (***t***-**Bu**-**G**_**2**_ **N**)_**2**_, (***t***-**Bu**-**G**_**1**_ **N**)_**2**_ and **CC**(***t***-**Bu**-**G**_**1**_ **N**)_**3**_.

The dendrons with *tert*-butyl amide groups on peripheral benzene were prepared according to our previous procedure (Figure S1)^71–76^. Dendrimers (***t***-**Bu**-**G**_**2**_ **N**)_**2**_, **CC**(***t***-**Bu**-**G**_**1**_ **N**)_**3**_ and (***t***-**Bu**-**G**_**1**_ **N**)_**2**_ were synthesized as summarized in Fig. 2. The dendrimer (***t***-**Bu**-**G**_**1**_ **N**)_**2**_ was prepared by reacting ***t***-**Bu**-**G**_**1**_ **Cl** with ***t***-**Bu**-**G**_**1**_ **NH** in the presence of K_2_CO_3_ at 170 °C in a sealed tube. In a similar manner, (***t***-**Bu**-**G**_**2**_ **N**)_**2**_ and **CC**(***t***-**Bu**-**G**_**1**_ **N**)_**3**_ were prepared by reacting ***t***-**Bu**-**G**_**2**_ **Cl** with ***t***-**Bu**-**G**_**2**_ **NH** and ***t***-**Bu**-**G**_**1**_ **NH**, respectively. Dendrimers (***t***-**Bu**-**G**_**n**_ **N**)_**2**_ (n = 1, 2) and **CC**(***t***-**Bu**-**G**_**1**_ **N**)_**3**_ were further characterized by ^1^H and ^13^C NMR spectroscopy and mass spectrometry. The R_1_ moiety of the dendrons, containing the *1*,*3*,*5*-triamidobenzene (*1*-*3*-*5*-TAB) moiety in constructing bulky dendritic frameworks will be discussed later.

**Figure 3:**
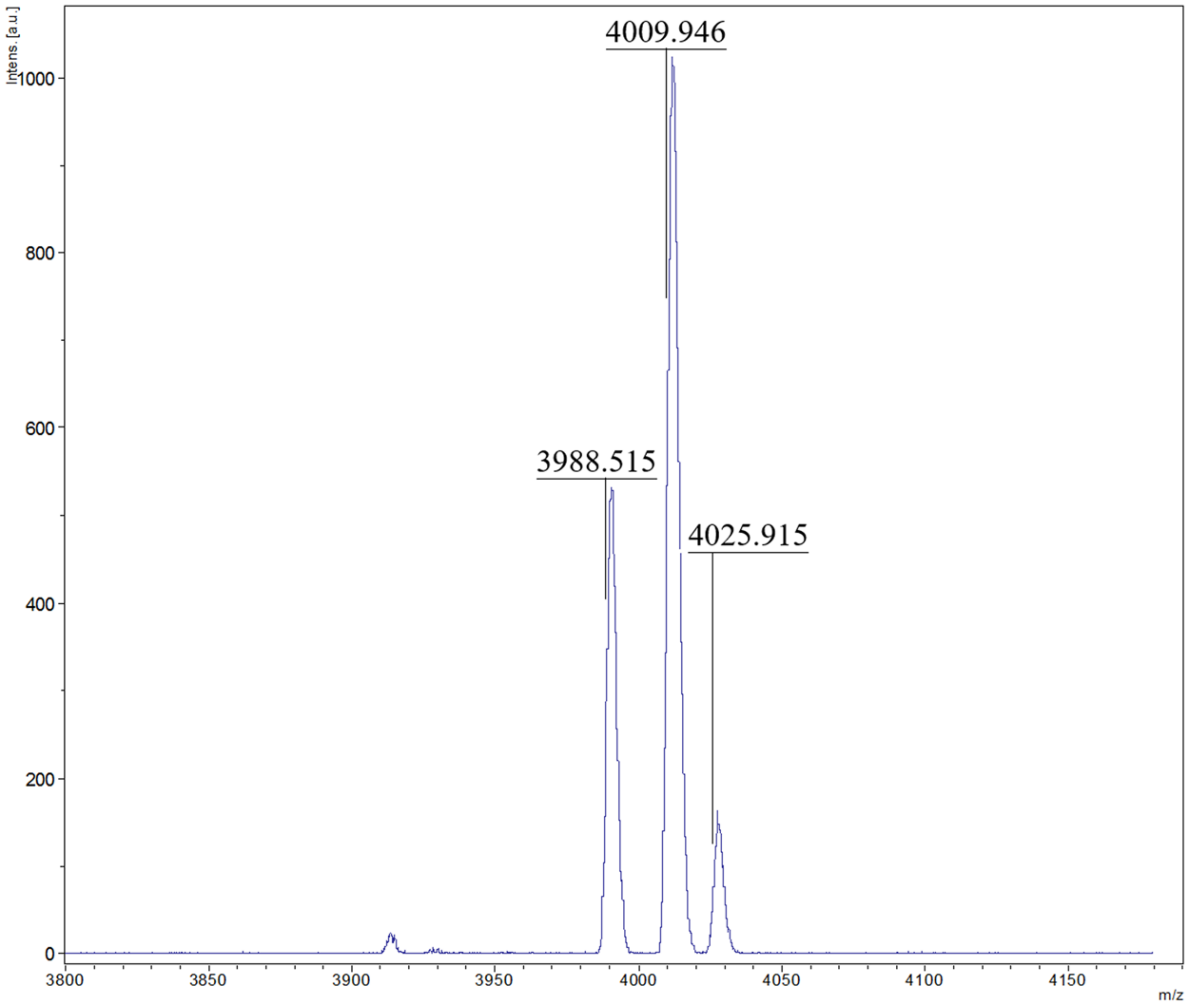
The mass spectrum of (***t***-**Bu**-**G**_**2**_ **N**)_**2**_ obtained by MALDI-TOF.

As a representative example for these dendrimers, the mass spectrum of (***t***-**Bu**-**G**_**2**_ **N**)_**2**_ is shown in Fig. 3 where the peaks corresponding to [M]^+^, [M + Na−H]^+^ and [M + K−H]^+^ at m/z of 3988.5, 4009.9 and 4025.9, respectively, are clearly observed. All three dendrimers of this study contain many amide moieties and, therefore, easily adsorb moisture from the surroundings, as confirmed by microanalysis. The differences between calculated and experimental percentages of C, H and N for (***t***-**Bu**-**G**_**2**_ **N**)_**2**_·10H_2_O are within 0.1%. The TGA analysis indicates that the bulk (***t***-**Bu**-**G**_**2**_ **N**)_**2**_ starts to decompose at ~400 °C, and the residue of ~28% still remains at 800 °C. The TGA studies of (***t***-**Bu**-**G**_**1**_ **N**)_**2**_ and **CC**(***t***-**Bu**-**G**_**1**_ **N**)_**3**_ show similar results (Figure S3). Thus the dendritic molecules show relatively high thermal stability.

**Figure 4:**
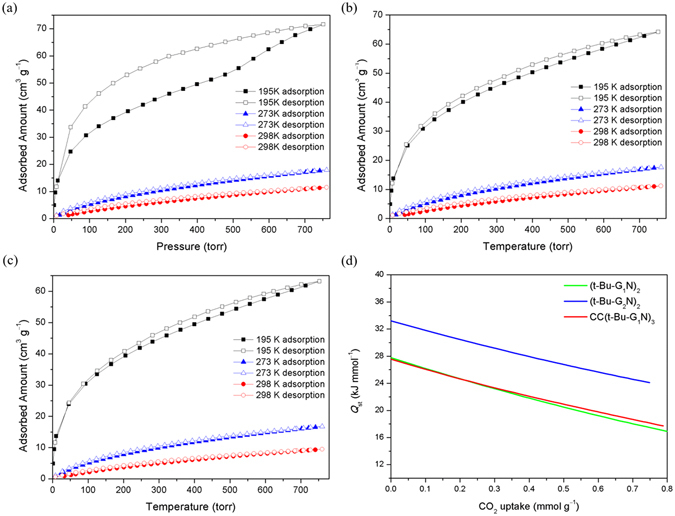
(**a**) CO_2_ sorption isotherms for (***t***-**Bu**-**G**_**1**_ **N**)_**2**_ measured at 195 K, 273 K and 298 K. (**b**) CO_2_ sorption isotherms for **CC**(***t***-**Bu**-**G**_**1**_ **N**)_**3**_ measured at 195 K, 273 K and 298 K. (**c**) CO_2_ sorption isotherms for (***t***-**Bu**-**G**_**2**_ **N**)_**2**_ measured at 195 K, 273 K and 298 K. (**d**) Isosteric heat (*Q*_st_) of CO_2_ sorption for (***t***-**Bu**-**G**_**2**_ **N**)_**2**_, **CC**(***t***-**Bu**-**G**_**1**_ **N**)_**3**_ and (***t***-**Bu**-**G**_**1**_ **N**)_**2**_ (only sorption data at 273 K and 298 K were used).

The CO_2_ sorption behavior of (***t***-**Bu**-**G**_**1**_ **N**)_**2**_, **CC**(***t***-**Bu**-**G**_**1**_ **N**)_**3**_ and (***t***-**Bu**-**G**_**2**_ **N**)_**2**_ was studied to determine their surface areas and the heats of sorption of CO_2_. All dendrimers were degassed at 125 °C for 1 day under high vacuum to remove water molecules and then their gas sorption properties were studied (Fig. 4a–c). The CO_2_ sorption isotherms for each of (***t***-**Bu**-**G**_**1**_ **N**)_**2**_, **CC**(***t***-**Bu**-**G**_**1**_ **N**)_**3**_ and (***t***-**Bu**-**G**_**2**_ **N**)_**2**_ were measured at 195, 273 and 298 K. The gas sorption by a microporous material is an exothermal process^77–82^, and therefore the amount of CO_2_ adsorbed by the dendrimers increases at lower temperature. The CO_2_ sorption by (***t***-**Bu**-**G**_**1**_ **N**)_**2**_, **CC**(***t***-**Bu**-**G**_**1**_ **N**)_**3**_ and (***t***-**Bu**-**G**_**2**_ **N**)_**2**_ at 195 K is in the range of 63~72 cm^3^/g (Fig. 4a–c). The Brunauer-Emmett-Teller (BET) surface areas of (***t***-**Bu**-**G**_**1**_ **N**)_**2**_, **CC**(***t***-**Bu**-**G**_**1**_ **N**)_**3**_ and (***t***-**Bu**-**G**_**2**_ **N**)_**2**_ were calculated to be ~136.0 m^2^/g, ~138.2 m^2^/g, and ~135.9 m^2^/g, respectively, and their Langmuir surface areas were estimated to be ~214.4 m^2^/g, ~218.3 m^2^/g, and ~215.4 m^2^/g, respectively.

Compared with those of **CC**(***t***-**Bu**-**G**_**1**_ **N**)_**3**_ and (***t***-**Bu**-**G**_**2**_ **N**)_**2**_, the sorption behavior of (***t***-**Bu**-**G**_**1**_ **N**)_**2**_ is different, which may be a result of its lower molecular weight. It is possible that the CO_2_ gas pressure increases the amount of void space in the bulk by expanding the existing voids or generating new ones. The sorption behavior changes around 500 torr at 195 K (Fig. 4a). No such phenomenon was observed for **CC**(***t***-**Bu**-**G**_**1**_ **N**)_**3**_ or (***t***-**Bu**-**G**_**2**_ **N**)_**2**_, presumably because of their higher molecular weight and a higher energy to expand their 3D frameworks.

To better understand their sorption capacity, the isosteric heats of CO_2_ sorption (*Q*_st_) of (***t***-**Bu**-**G**_**1**_ **N**)_**2**_, **CC**(***t***-**Bu**-**G**_**1**_ **N**)_**3**_ and (***t***-**Bu**-**G**_**2**_ **N**)_**2**_ were calculated from the isotherms by the virial method (using data at 273 K and 298 K; Figures S5 and S6)^79, 80^. As shown in Fig. 4d, the isosteric heat (*Q*_st_) for (***t***-**Bu**-**G**_**2**_ **N**)_**2**_ was found to be 33.2 kJ/mol at zero coverage which is higher than the corresponding *Q*_st_ of (***t***-**Bu**-**G**_**1**_ **N**)_**2**_ (27.8 kJ/mol) and **CC**(***t***-**Bu**-**G**_**1**_ **N**)_**3**_ ﻿(27.6 kJ/mol), respectively. The isosteric heats (*Q*_st_) of these compounds are, to some extend, higher than those of most organic materials in the literature, presumably due to an azaheterocycle in the dendritic molecule^43, 46–48^.

**Figure 5:**
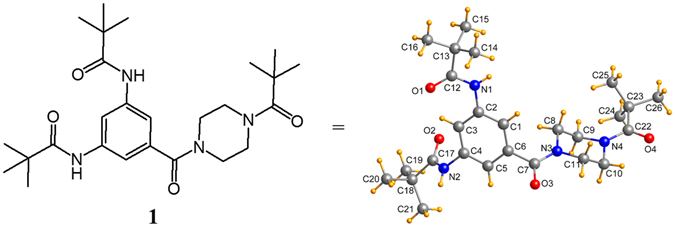
The molecular structure and conformation of **1** in the studied crystal.

**Figure 6:**
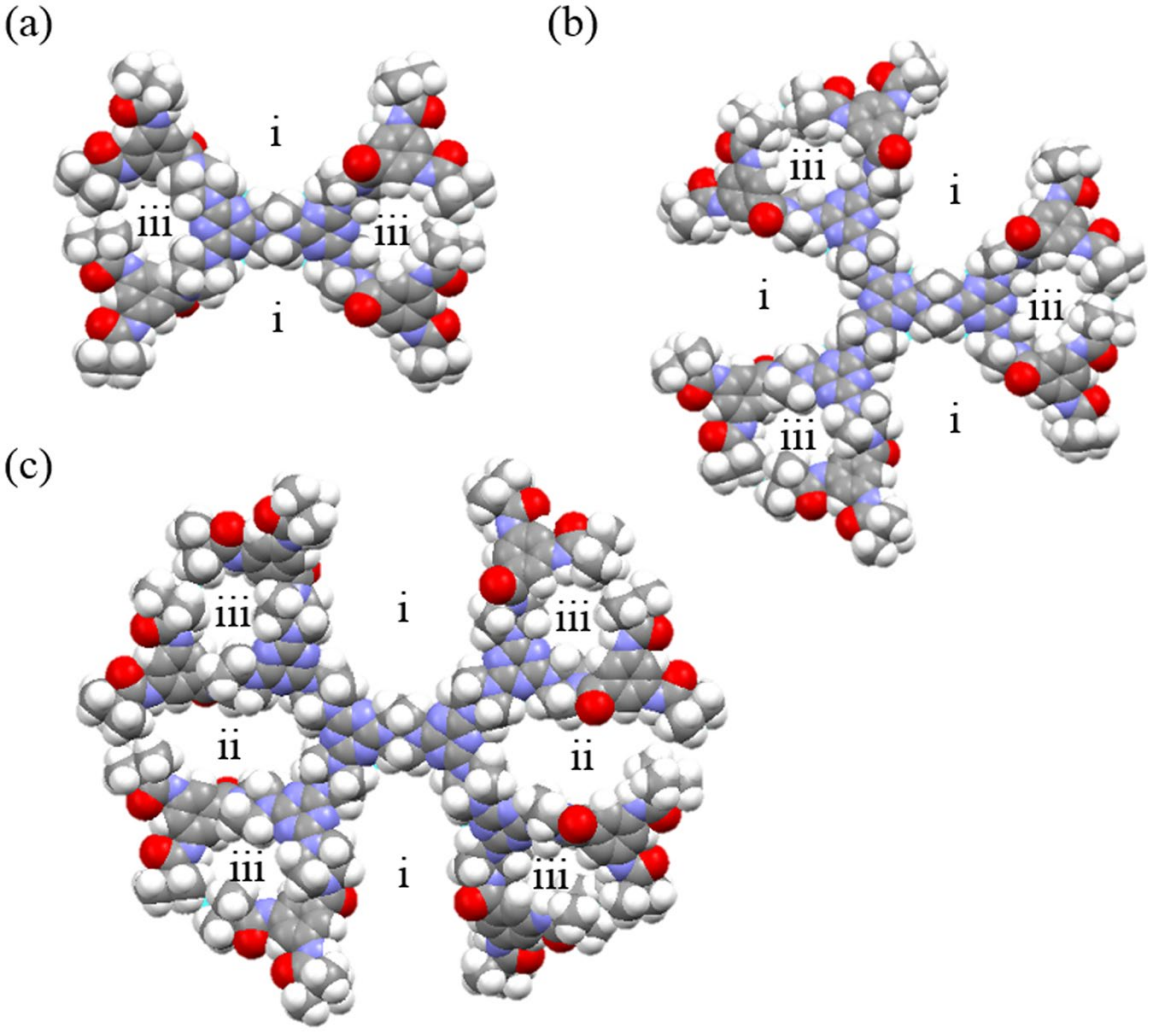
The equilibrium molecular conformations of (**a**) (***t***-**Bu**-**G**_**1**_ **N**)_**2**_, (**b**) **CC**(***t***-**Bu**-**G**_**1**_ **N**)_**3**_, (**c**) (***t***-**Bu**-**G**_**2**_ **N**)_**2**_ shown as space-fill models (N: purple, O: red, C: gray, H: white). Potential sorption sites are labelled with small Roman letters.

It is interesting that these three dendrimers display similar BET surface areas in spite of different molecular weights, while (***t***-**Bu**-**G**_**2**_ **N**)_**2**_ shows higher *Q*_st_ than (***t***-**Bu**-**G**_**1**_ **N**)_**2**_ and **CC**(***t***-**Bu**-**G**_**1**_ **N**)_**3**_. To understand this phenomenon, we attempted to investigate the 3D stacking of the molecules in the solid. Although the dendrimers themselves could not be obtained as single crystals, compound **1**, representing the peripheral moiety of the studied dendrimers, was crystallized and studied by the single crystal X-ray diffraction method (Fig. 5 and Table S1). Based on this crystal structure analysis study, the oxygen atoms of both *t*-butylamides in compound **1** are directed towards each other. This geometry was taken as a starting conformation of the R_**1**_ moiety for computational studies (Fig. 6).

The optimized conformation of the peripheral moiety of dendrimers in the gas phase was obtained by the CaChe program using MM2 model. The conformation of ***t***-**Bu**-**G**_**1**_ **Cl** was established using two optimized R_**1**_ units and the planar triazine, and then optimized. The conformation of ***t***-**Bu**-**G**_**1**_ **NH** was established using one optimized ***t***-**Bu**-**G**_**1**_ **Cl** and piperazine in chair form, and then optimized. In a similar manner, the optimized conformations of ***t***-**Bu**-**G**_**2**_ **Cl** and ***t***-**Bu**-**G**_**2**_ **NH** were obtained. The conformation of (***t***-**Bu**-**G**_**1**_ **N**)_**2**_ was obtained by combining one optimized ***t***-**Bu**-**G**_**1**_ **Cl** and one optimized ***t***-**Bu**-**G**_**1**_ **NH**, and then optimized. The optimized conformations of (***t***-**Bu**-**G**_**2**_ **N**)_**2**_ and **CC**(***t***-**Bu**-**G**_**1**_ **N**)_**3**_ were obtained accordingly (Fig. 6). Although the computing simulations were completed in the gas phase and the optimized conformations of dendrimers may differ from those in the bulk solids, the results help to understand the reason why the three dendrimers display different *Q*_st_ and to model possible dendritic frameworks using the crystal structure data for compound **1** as well as the FT-IR and powder-XRD results for (**t**-**Bu**-**G**_**1**_ **N**)_**2**_ discussed later.

**Figure 7:**
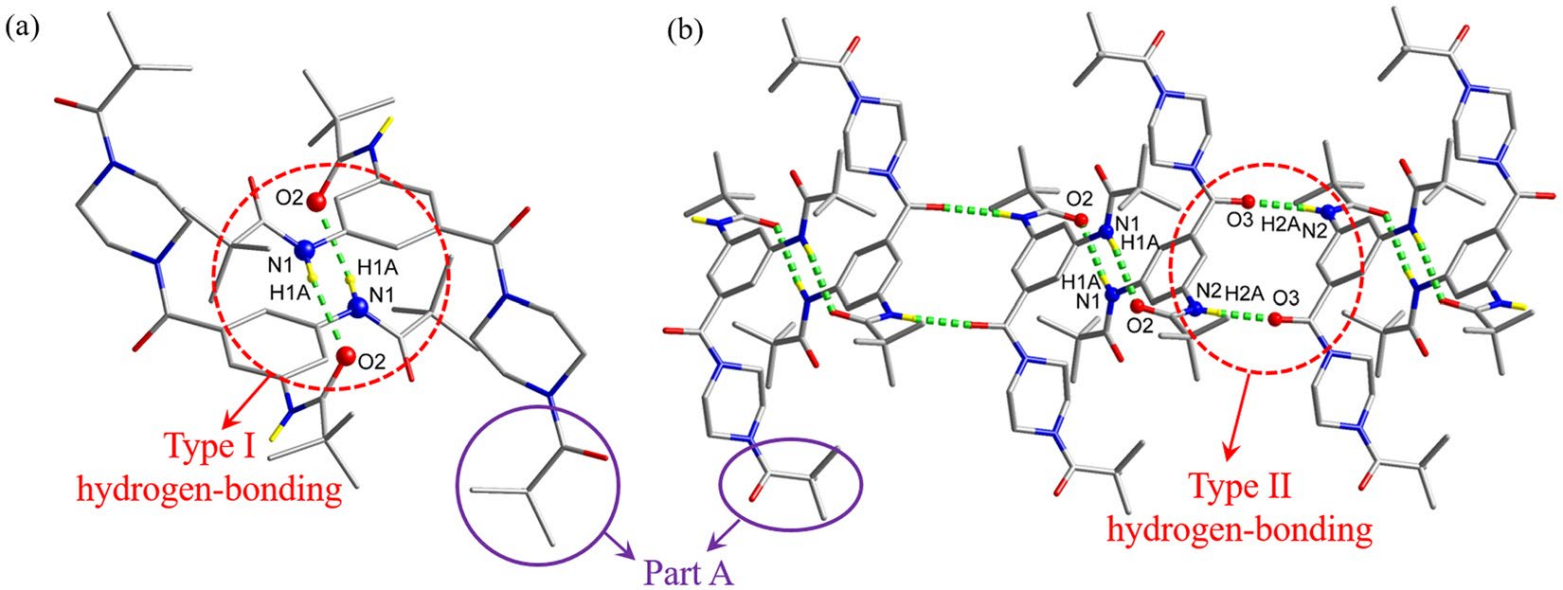
(**a**) Type I H-bond interaction and (**b**) type II H-bond interaction in **1**.

To reasonably establish the molecular packing of (***t***-**Bu**-**G**_**1**_ **N**)_**2**_, **CC**(***t***-**Bu**-**G**_**1**_ **N**)_**3**_, and (***t***-**Bu**-**G**_**2**_ **N**)_**2**_ in the bulk dendrimers, the intermolecular interaction of **1** in the crystal structure was employed as a model. The amide groups of **1** form two types of strong H-bonds (N−H···O); one is at O2···H1A (N1), and the other is at O3···H2 (N2). Two H-bonds of type I (distance: ~2.97 Å for O···N and angle: 157.3° for N−H···O), allow two molecules to form a dimer (Fig. 7a). Two H-bonds of type II (distance: ~2.84 Å for O···N and angle: 160.0° for N−H···O), link the dimers into a 1D polymer (Fig. 7b). Surprisingly, the amide groups in part A region are not involved in any H-bond interactions suggesting that *1*,*3*,*5*-triamidobenzene (*1*-*3*-*5*-TAB) block does not enter into the interstitial void space of neighboring dendritic molecules due to the strong H-bond interaction between the *1*-*3*-*5*-TAB blocks.

**Figure 8:**
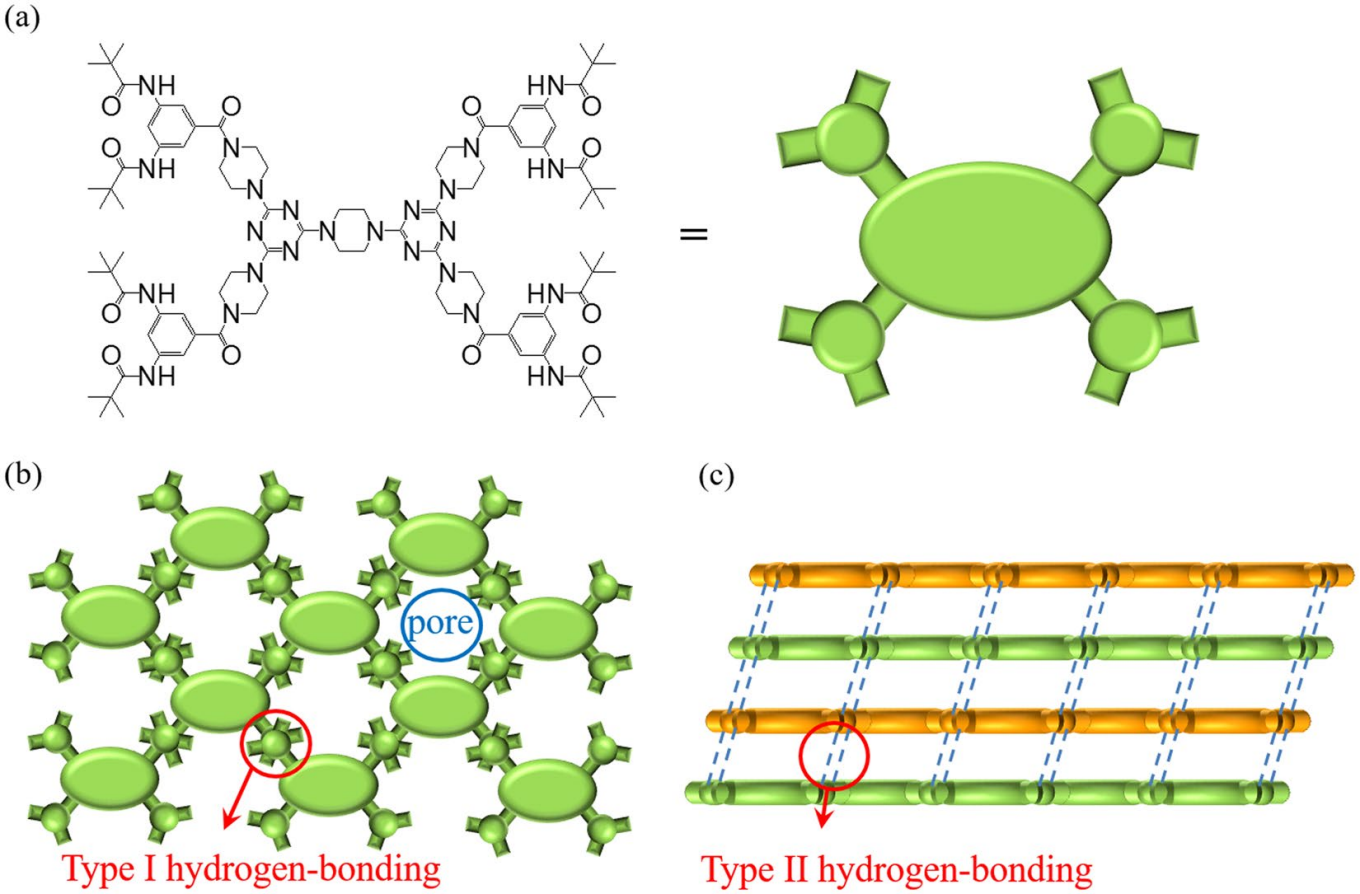
(**a**) (***t***-**Bu**-**G**_**1**_ **N**)_**2**_ as a supramolecular building unit with four branches, (**b**) the 2D network from (***t***-**Bu**-**G**_**1**_ **N**)_**2**_ units connected by H-bonds of type I, and (**c**) the 3D framework formed from 2D networks by H-bonds of type II. The scheme illustrates supramolecular connectivity of the units of (***t***-**Bu**-**G**_**1**_ **N**)_**2**_ in the solid state rather than the real 3D geometry of the dendrimer.

**Figure 9:**
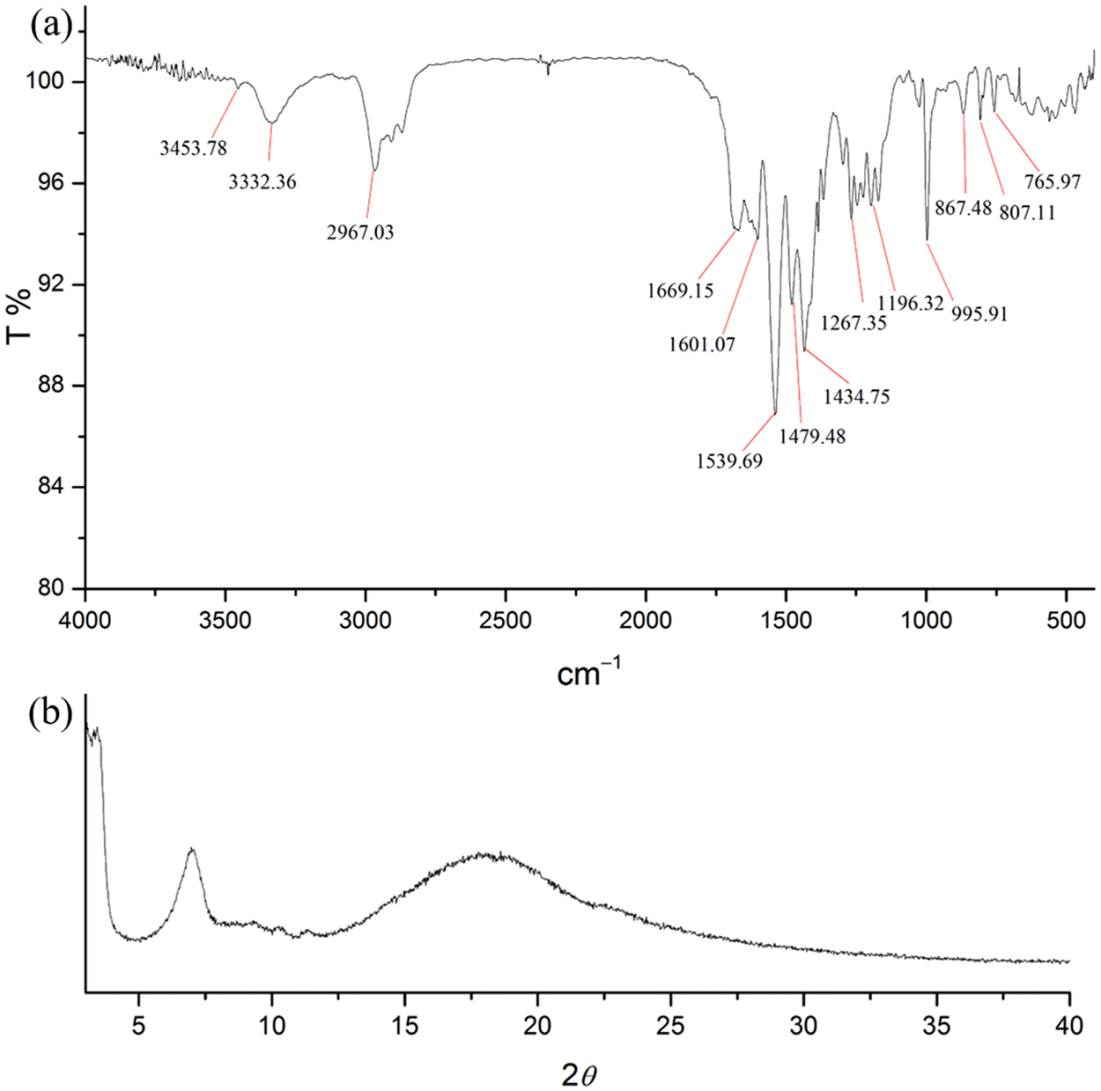
The FT-IR spectrum (**a**) and powder-XRD pattern (**b**) of bulk (***t***-**Bu**-**G**_**1**_ **N**)_**2**_.

**Figure 10:**
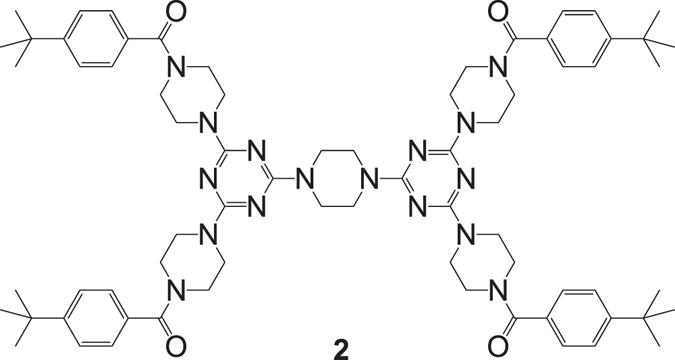
Structure of **2**.

Based on the simulation, the molecules of (***t***-**Bu**-**G**_**2**_ **N**)_**2**_, **CC**(***t***-**Bu**-**G**_**1**_ **N**)_**3**_ and (***t***-**Bu**-**G**_**1**_ **N**)_**2**_ can be approximated as supramolecular building units with different shape and intermolecular connectivity. The peripheral amides of dendrimers construct porous dendritic frameworks through intermolecular H-bonds between the units. The dendrimer (***t***-**Bu**-**G**_**1**_ **N**)_**2**_ is used here as an example to demonstrate a possible stacking mode. As shown in Fig. 8a, the peripheral part of (***t***-**Bu**-**G**_**1**_ **N**)_**2**_ bears a partial structure of compound **1**. Thus, it is reasonable to assume that the dendrimer (***t***-**Bu**-**G**_**1**_ **N**)_**2**_ also possesses the H-bond interaction in the solid state as found in **1**. According to the literature, the free N-H stretching and H-bond N-H stretching of the amide moiety arises at about 3444 cm^−1^ (weak shoulder) and 3310 cm^−1^ (very broad), respectively^83, 84^. On the FT-IR spectrum of (***t***-**Bu**-**G**_**1**_ **N**)_**2**_, the corresponding weak stretching at about 3453 cm^−1^ and very broad stretching at about 3332 cm^−1^ are observed in the solid state (Fig. 9a). Based on these data, the percentage ratio of the free N-H stretching to H-bond N-H stretching is less than 5% and thus it is reasonable to assume that the free N-H moiety would not affect the gas sorption behavior significantly. According to the above observations, we may simplify the structural skeleton of (***t***-**Bu**-**G**_**1**_ **N**)_**2**_ as a rigid unit with four branches; each branch can form four strong H-bond interactions, two H-bonds of type I and two H-bonds of type II as discussed above (Fig. 7). The simplified skeleton may not account for all the details of the 3D structure, but it illustrates how the molecules of (***t***-**Bu**-**G**_**1**_ **N**)_**2**_ are likely to form a 2D network by the H-bonds of type I and a 3D framework subsequently constructed by the H-bonds of type II linking the 2D networks (Fig. 8b and c). It should be noted that the skeleton framework shown in Fig. 8b or Fig. 8c illustrates supramolecular connectivity of the units of (***t***-**Bu**-**G**_**1**_ **N**)_**2**_ in the solid state rather than the real 3D geometry of the dendrimer. The powder-XRD pattern of (***t***-**Bu**-**G**_**1**_ **N**)_**2**_ (Fig. 9b) shows very broad peaks indicating the dendrimer does not crystallize easily, similar to some porous COFs reported in the literature^53–55^. The powder-XRD pattern of **2** (Fig. S7b), however, shows sharper reflection peaks as compound **2** (Fig. 10) cannot form strong H-bonds to establish a 3D framework. It can be reasonably assumed that (***t***-**Bu**-**G**_**1**_ **N**)_**2**_ forms porous framework without allowing the peripheral amides to imbed in the void space of adjacent dendritic molecules because the molecules are fixed tightly in a regular order by strong intermolecular H-bonds of types I and II.

As shown in this work, the BET surface areas of the three dendrimers, all constructed by the piperazine and triazine moieties, are similar to each other (in the range of 136–138 m^2^/g). A previously reported COF from the same constructing units (piperazine and triazine) has a BET area of ~165 m^2^/g^50^. The difference in the BET surface areas of the dendritic and covalent organic frameworks may be defined by the connecting fragments. As demonstrated in Fig. 6, each dendrimer contains, at least, the open holes (type i) and the closed voids (type iii). However, the closed voids of type iii may be less accessible to the molecules of gas due to the steric hindrance of the peripheral *tert*-butyl groups constructing the bulky dendritic frameworks by strong H-bond interaction between *1*-*3*-*5*-TAB blocks (Fig. 8). This may explain why the BET surface area of the COF reported in the literature is somewhat higher than those of the studied dendrimers^50^. Therefore, the effective gas sorption in the bulk dendrimers should possibly start at the open hole of type i and the closed void of type ii (Fig. 6) as further discussed later. Although the size of open hole i in each dendrimer is different and dendrimer (***t***-**Bu**-**G**_**2**_ **N**)_**2**_ additionally contains the closed pore ii, the total void space in the bulky stacking of the three dendritic frameworks for gas adsorption should be very similar because all the void space, constructed by the triazine and piperazine units, is not effected by the peripheral amide moieties. To further verify this assumption, dendrimer **2** (Fig. 10), having similar connecting species but different peripheral moieties than (***t***-**Bu**-**G**_**1**_ **N**)_**2**_, was prepared and studied, and it was found to have a low sorption ability; the sorption at 195 K is only 24 cm^3^/g and the BET surface area is 47 m^2^/g (Figure S8). The isosteric heats of CO_2_ sorption (*Q*_st_) could not be calculated for **2** due to very poor sorption at 273 K and 298 K (Figure S8). Without the strong H-bond interaction to fix the dendritic unit as discussed above (Fig. 7), the void space of **2** in the bulky stacking was significantly reduced presumably due to the imbedding of peripheral moieties of adjacent molecules into the space.

Interestingly, the isosteric heats of CO_2_ sorption (*Q*_st_) for (***t***-**Bu**-**G**_**2**_ **N**)_**2**_ are higher than those for **CC**(***t***-**Bu**-**G**_**1**_ **N**)_**3**_ and (***t***-**Bu**-**G**_**1**_ **N**)_**2**_, possibly due to the closed voids of type ii, because the smaller closed void space in (***t***-**Bu**-**G**_**2**_ **N**)_**2**_ can be responsible for stronger attraction of a single CO_2_ molecule. This observation also supports our previous assumption that the closed voids of type iii are hardly accessible to gas, otherwise the *Q*_st_ for (***t***-**Bu**-**G**_**2**_ **N**)_**2**_ should not have been very different from those for the other dendrimers as all the three have closed voids of type iii.

The (***t***-**Bu**-**G**_**2**_ **N**)_**2**_ dendrimer consisting of effective closed and open void spaces in each single molecule should be a good material for catalytic reactions, because the closed void is a good adsorption site for CO_2_ gas, and the open hole may be a suitable sorption site for other reactants, either in the solid state or in solution.

## Conclusions

In brief, we have prepared a series of dendrimers containing piperazine and triazine as connecting units and *3*,*5*-di-*tert*-butylamidobenzene as a peripheral moiety. These dendrimers form supramolecular frameworks by strong H-bond interactions and their void space in the bulky stacking is controllable, as the peripheral amides do not imbed in the interstitial space of adjacent dendritic molecules. In other words, it may be possible to adjust the void space of the bulky dendritic frameworks by changing the corresponding connecting fragments of the dendrimer. In addition, one of the dendrimers possesses closed void for CO_2_ adsorption and exhibits a higher *Q*_st_ than others, which may be utilized to facilitate catalytic reactions where two types of void space can efficiently accommodate two reactants to bring them close for the reaction. Dendrimers can be readily prepared on a large scale and easily purified. The triazined-based dendrimers, displaying good solubility and chemical stability, may become convenient as porous materials for various applications, such as for separation of N_2_ - CO_2_ gas mixtures or the development of catalytic reactions.

## Methods

The synthesis of compound **A**, for preparing dendrons ***t***-**Bu**-**G**_**1**_ **Cl**, ***t***-**Bu**-**G**_**1**_ **NH**, ***t***-**Bu**-**G**_**2**_ **Cl**, and ***t***-**Bu**-**G**_**1**_ **NH**, and all characterization data were described in Supporting Information.

### The general procedure for dendrimers

***t***-**Bu**-**G**_**n**_ **Cl** (1 mmol; n = 1 or 2) and ***t***-**Bu**-**G**_**n**_ **NH** (1 mmol; n = 1 or 2), prepared according to our previous procedure^71–75^, were dissolved in dry THF (20 mL) in a sealed tube. Potassium carbonate (0.5 g, 5 mmol) was then added and sealed. The resulting mixture was heated at 170 °C for 72 hour. Water (20 mL) was added to the mixture and the solution was extracted with CH_2_Cl_2_ (50 mL × 2). The combined extracts were washed with water (20 mL), dried over MgSO_4_ and concentrated at reduced pressure. The residue was further recrystallized from CH_2_Cl_2_-CH_3_OH (1:20) to give the pure desired dendrimer.

(**t**-**Bu**-**G**_**1**_ **N**)_**2**_ was prepared in 83.4% yield. ^1^H-NMR (300 MHz, DMSO-d_6_, 25 °C, TMS): δ = 1.21 (s, 72 H, 24 × CH_3_), 3.72 + 3.63 (br 2 s, 40 H, 20 × CH_2_), 7.40 (s, 8 H, 8 × Ar-H), 8.09 (s, 4 H, 4 × Ar-H), 9.29 ppm (s, 8 H, 8 × NH); ^13^C-NMR (300 MHz, DMSO-d_6_, 25 °C, TMS): δ = 27.14, 42.68, 113.88, 135.73, 139.43, 164.73, 168.99, 176.61 ppm; MS: M/Z: calcd for C_94_H_131_N_24_O_12_ (M^+^): 1790.2; found: 1790.9; elemental analysis: calcd (%) for (C_94_H_132_N_24_O_12_ + 7H_2_O) C 58.92, H 7.68, N 17.54; found: C 58.72, H 7.51, N 17.42.

(**t**-**Bu**-**G**_**2**_ **N**)_**2**_ was prepared in 69.3% yield. ^1^H-NMR (300 MHz, DMSO-d_6_, 25 °C, TMS): δ = 1.21 (s, 144 H, 48 × CH_3_), 3.75 + 3.42 (br 2 s, 104 H, 52 × CH_2_), 7.42 (s, 16 H, 16 × Ar-H), 8.09 (s, 8 H, 8 × Ar-H), 9.30 ppm (s, 16 H, 16 × NH); ^13^C-NMR (300 MHz, DMSO-d_6_, 25 °C, TMS): δ = 27.14, 41.86, 42.68, 47.21, 113.55, 113.93, 135.71, 139.43, 164.77, 169.03, 176.61 ppm; MS: M/Z: calcd for C_206_H_287_N_60_O_24_Na (M + Na-H)^+^: 4010.9; found: 4009.9; elemental analysis: calcd (%) for (C_206_H_288_N_60_O_24_ + 10H_2_O) C 59.35, H 7.45, N 20.16; found: C 59.45, H 7.50, N 20.18.

**CC**(**t**-**Bu**-**G**_**1**_ **N**)_**3**_ was prepared in 63.8% yield. ^1^H-NMR (300 MHz, DMSO-d_6_, 25 °C, TMS): δ = 1.22 (s, 108 H, 36 × CH_3_), 3.75 + 3.40 (br 2 s, 72 H, 36 × CH_2_), 7.42 (s, 12 H, 12 × Ar-H), 8.10 (s, 6 H, 6 × Ar-H), 9.36 ppm (s, 12 H, 12 × Ar-H); ^13^C-NMR (300 MHz, DMSO-d_6_, 25 °C, TMS): δ = 27.45, 41.88, 43.00, 47.47, 113.89, 114.24, 136.03, 139.75, 165.09, 169.33, 176.92 ppm; MS: M/Z: calcd for C_150_H_209_N_42_O_18_Na (M + Na−H)^+^: 2911.5; found: 2911.3; elemental analysis: calcd (%) for (C_150_H_210_N_42_O_18_ + 8H_2_O) C 59.39, H 7.51, N 19.39; found: C 59.28, H 7.63, N 19.20.

### Materials and Instruments

Thermogravimetric analyses were performed under nitrogen with a Perkin-Elmer TGA-7 TG analyzer. Elemental analyses were conducted with a Perkin-Elmer 2400 CHN elemental analyzer. Infrared spectra were recorded in the range of 4000–400 cm^−1^ with a Frontier FT-IR spectrometer. Powder X-ray diffraction measurements were performed at room temperature on a Bruker D8 Advance diffractometer with a copper radiation source with a step size of 0.02° in *θ* and a scan speed of 1 s per step size. Brunauer−Emmett−Teller analyses were investigated with a Micrometrics ASAP 2020 system using carbon dioxide as the adsorbate at 195 K, 273 K, and 298 K.

### Crystal Structure Determination

A suitable single crystal of **1** with dimensions of 0.40 × 0.35 × 0.24 mm^3^ was mounted on the tip of a glass fiber and placed onto the goniometer head for unit cell measurement and intensity data collection using a Bruker APEX-II CCD diffractometer with graphite-monochromatized Mo Kα radiation (λ = 0.71073 Å). Collection of intensity data was conducted at 155 K. Empirical absorptions correction was applied using the multiscan method. The structure was solved by direct methods and refined against *F* ^*2*^ by the full-matrix least-squares technique using the WINGX and SHELX-97 software packages. Anisotropical displacement parameters were assigned to non-hydrogen atoms. Carbon-bound hydrogen atoms were placed in calculated positions and refined using a riding model. Nitrogen-bound hydrogen atoms were located on the difference Fourier map and refined using a riding model. Isotropic thermal factors of all hydrogen atoms were derived from the parent atoms. Experimental details for X-ray data collection and the refinement are summarized in Table S1.

## Electronic supplementary material

Electronic Supporting Information
