# Supplementary material for: Design of a Peripheral Building Block for H-Bonded Dendritic Frameworks and Analysis of the Void Space in the Bulk Dendrimers
Source: Sci Rep. 2017 Jun 16;7:3649. doi: 10.1038/s41598-017-03684-y (PMC5473840; doi:10.1038/s41598-017-03684-y)
Supplement: Supplementary file 1 — Electronic Supporting Information [file 41598_2017_3684_MOESM1_ESM.pdf]

Electronic Supporting Information

Design of a Peripheral Building Block for H-Bonded Dendritic Frameworks and Analysis of the Void Space in the Bulk Dendrimers

Cheng-Hua Lee,[a,b,c] Dmitriy V. Soldatov,[a] Chung-Hao Tzeng,[b] Long-Li Lai[b] & Kuang-Lieh Lu[c]*

[a]Department of Chemistry, University of Guelph, Guelph, Ontario, N1G 2W1, Canada

[b]Department of Applied Chemistry, National Chi Nan University, 1 Daxue Rd., Puli, Nantou County 545, Taiwan, E-mail: lilai@ncnu.edu.tw

[c]Institute of Chemistry, Academia Sinica, Taipei 115, Taiwan

Experiment Section:

S1. Preparation of dendrons

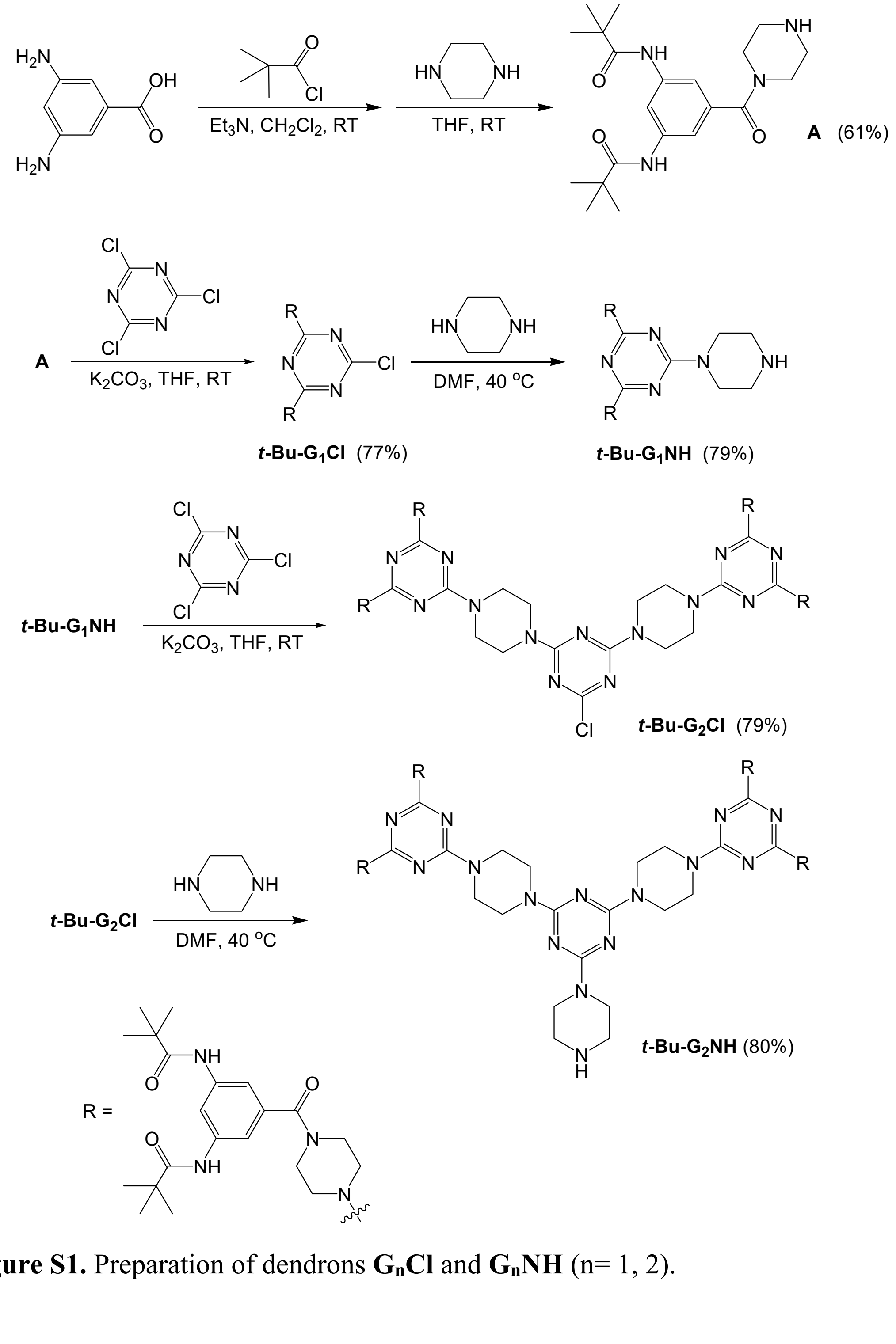

Figure S1. Preparation of dendrons G_nCl and G_nNH (n= 1, 2).

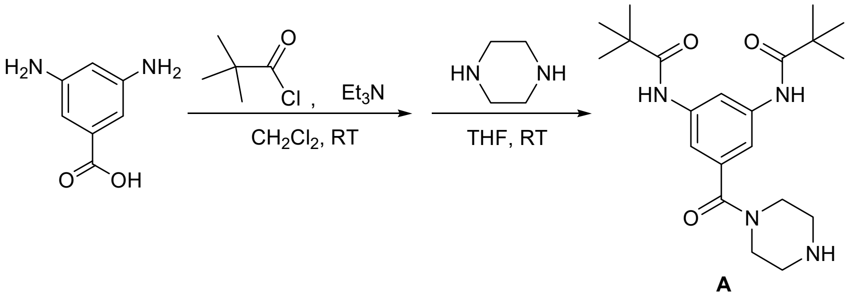

3,5-Diaminobenzoic acid (4.6 g, 30 mmol) and triethylamine (27.3 g, 270 mmol) were added to dried CH_2Cl_2 (30 mL) in an ice bath. Pivaloyl chloride (18.3 mL, 150 mmol) was added and the resulting mixture was stirred at 5 °C for 1 hr. Dried CH_2Cl_2 (150 mL) was then added and the resulting solution was stirred at room temperature for 12 h. The solution was washed with aqueous K_2CO_3 solution (21 g in 200 mL water), and then washed with water (50 mL × 2). The solution was dried over $MgSO_4$ and evaporated at reduced pressure to give a white solid, which was then dissolved in dried THF (150 mL) in an ice-bath. Piperazine (10.34 g, 120 mmol) was then added, and the solution was stirred at room temperature for 3 h. After the reaction, the solution was washed with aqueous K_2CO_3 solution (5 g in 50 mL water), and then washed with water (50 mL). The solution was dried over $MgSO_4$ and evaporated at reduced pressure. The residue was purified by chromatography on silica (eluent: THF - MeOH in 1:1 ratio) and then recrystallized from THF - hexane (1:10) to give compound **A** in 60.8% yield (2.74 g). ^{1}H-NMR (300 MHz, $CDCl_3$, 25 °C, TMS): δ = 1.28 (s, 18H, 6×CH_3), 2.82+2.91 (br 2s, 4H, 2×CH_2), 3.45+3.73 (br 2s, 4H, 2×CH_2), 7.37 (s, 2H, Ar-H), 7.62 (s, 2H, 2×NH), 7.93 ppm (s, 1H, Ar-H); ^{13}C-NMR (300 MHz, $CDCl_3$, 25 °C, TMS): δ = 27.54, 39.70, 43.35, 45.93, 46.49, 49.07, 113.28, 114.35, 136.26, 138.83, 169.89, 177.31 ppm; MS: M/Z: calcd for $C_{21}H_{32}N_4O_3Na$ $(M+Na)^+$: 411.5; found: 411.2; elemental analysis: calcd (%) for ($C_{21}H_{32}N_4O_3$+0.3H_2O) C 64.03, H 8.34, N 14.22; found: C 64.11, H 8.34, N 13.94.

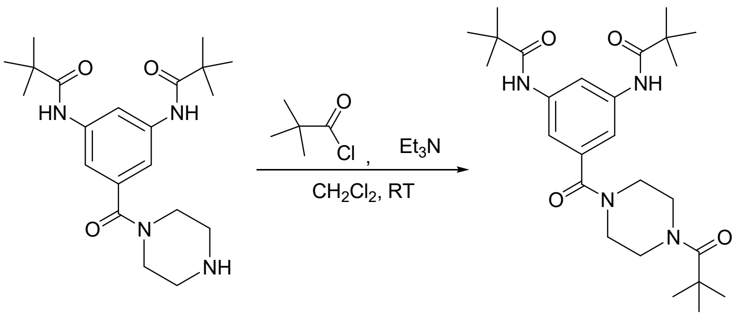

A **1**

Compound **A** (0.78 g, 2 mmol) and pivaloyl chloride (0.36 g, 3 mmol) were added to dried THF (10 mL) in an ice bath and stirred at 5 °C for 30 min. Triethylamine (0.61 g, 6 mmol) was then added and the resulting solution was stirred at room temperature for 12 h. The solution was washed with aqueous K_2CO_3 solution (0.83 g in 20 mL water) and then washed with water (20 mL × 2). The solution was dried over $MgSO_4$ and evaporated at reduced pressure to give a white solid. The residue was recrystallized from CH_2Cl_2 - Hexane (2:10) to give pure compound **1** in 90% yield (0.85 g). ^{1}H-NMR (300 MHz, $CDCl_3$, 25 °C, TMS): δ = 1.20 (s, 27H, 9×CH_3), 3.41+3.55+3.65 (br 3s, 8H, 4×CH_2), 7.31 (s, 2H, Ar-H), 7.81 (s, 1H, Ar-H), 8.00 ppm (s, 2H, 2×NH); ^{13}C-NMR (300 MHz, $CDCl_3$, 25 °C, TMS): δ = 27.43, 28.32, 38.68, 39.61, 42.28, 44.77, 45.33, 47.70, 113.47, 114.26, 135.57, 138.98, 170.10, 176.64, 177.30 ppm; MS: M/Z: calcd for $C_{26}H_{41}N_4O_4$ $(M+H)^+$: 473.6; found: 473.1; elemental analysis: calcd (%) for ($C_{26}H_{40}N_4O_4$) C 66.07, H 8.53, N 11.85; found: C 65.75, H 8.51, N 11.94.

All of the dendrons and dendrimers were prepared according to our previous procedures (Scheme S1). The reaction yields and characterization data for dendrons and dendrimers are as follows.

***t*-Bu-G$_1$Cl** was prepared in 76.7% yield. ^{1}H-NMR (300 MHz, DMSO-d$_6$, 25 °C, TMS): δ = 1.20 (s, 36H, 12×CH_3), 3.44-3.90 (m, 16H, 8×CH_2), 7.40 (s, 4H, 4×Ar-H), 8.07 (s, 2H, 2×Ar-H), 9.34 ppm (s, 4H, 4×NH); ^{13}C-NMR (300 MHz, DMSO-d$_6$, 25 °C, TMS): δ = 27.15, 40.35, 41.30, 42.96, 43.45, 46.80, 113.69, 113.98, 135.51, 139.47, 163.94, 168.90, 169.07, 176.63 ppm; MS: M/Z: calcd for $C_{45}H_{61}N_{11}O_6NaCl$ (M+Na-H)$^+$: 910.5; found: 910.7; elemental analysis: calcd (%) for ($C_{45}H_{62}N_{11}O_6Cl$+ 4H_2O) C 56.27, H 7.35, N 16.04; found: C 56.17, H 7.45, N 15.98.

***t*-Bu-G$_1$NH** was prepared in 78.6% yield. ^{1}H-NMR (300 MHz, DMSO-d$_6$, 25 °C, TMS): δ = 1.21 (s, 36H, 12×CH_3), 2.66 (s, 4H, 2×CH_2), 3.31 (s, 4H, 2×CH_2), 3.61+3.73 (br 2s, 16H, 8×CH_2), 7.39 (s, 4H, 4×Ar-H), 8.10 (s, 2H, 2×Ar-H), 9.30 (s, 4H, 4×NH) ppm; ^{13}C-NMR (300 MHz, DMSO-d$_6$, 25 °C, TMS): δ = 27.19, 41.66, 42.65, 43.15, 43.88, 45.52, 47.15, 113.63, 113.96, 135.79, 139.49, 164.67, 164.82, 169.10, 176.69 ppm; MS: M/Z: calcd for $C_{49}H_{71}N_{13}O_6Na$ (M+Na)$^+$: 961.2; found: 961.0; elemental analysis: calcd (%) for ($C_{49}H_{71}N_{13}O_6$+3H_2O) C 59.31, H 7.82, N 18.35; found: C 59.32, H 7.93, N 18.09.

***t*-Bu-G$_2$Cl** was prepared in 78.8% yield. ^{1}H-NMR (300 MHz, DMSO-d$_6$, 25 °C, TMS): δ = 1.20 (s, 72H, 24×CH_3), 3.62+3.74 (br 2s, 48H, 24×CH_2), 7.39 (s, 8H, 8×Ar-H), 8.07 (s, 4H, 4×Ar-H), 9.34 ppm (s, 8H, 8×NH); ^{13}C-NMR (300 MHz, DMSO-d$_6$, 25 °C, TMS): δ = 27.46, 41.99, 43.29, 47.45, 113.92, 114.26, 136.03, 139.76, 164.20, 165.07, 169.37, 176.96 ppm; MS: M/Z: calcd for $C_{101}H_{139}N_{29}O_{12}NaCl$ $(M+Na-H)^+$: 2009.8; found: 2010.3; elemental analysis: calcd (%) for ($C_{101}H_{140}N_{29}O_{12}Cl$+6$H_2O$) C 57.88, H 7.31, N 19.38; found: C 57.97, H 7.33, N 19.28.

***t*-Bu-G$_2$NH** was prepared in 79.8% yield (1.62 g). ^{1}H-NMR (300 MHz, DMSO-d$_6$, 25 °C, TMS): δ = 1.21 (s, 72H, 24×CH_3), 2.65 (s, 4H, 2×CH_2), 3.35 (s, 4H, 2×CH_2), 4.61+3.73 (br 2s, 48H, 24×CH_2), 7.40 (s, 8H, 8×Ar-H), 8.09 (s, 4H, 4×Ar-H), 9.34 (s, 8H, 8×NH) ppm; ^{13}C-NMR (300 MHz, DMSO-d$_6$, 25 °C, TMS): δ = 27.44, 41.88, 43.00, 44.11, 45.75, 47.34, 48.81, 114.23, 136.02, 139.74, 165.06, 169.33, 176.91 ppm; MS: M/Z: calcd for $C_{105}H_{149}N_{31}O_{12}Na$ $(M+Na)^+$: 2060.5; found: 2060.5; elemental analysis: calcd (%) for ($C_{105}H_{149}N_{31}O_{12}$+7$H_2O$) C 58.29, H 7.59, N 20.07; found: C 58.24, H 7.64, N 19.85.

(t-Bu-G$_1$N)$_2$ was prepared in 83.4 % yield. ^{1}H-NMR (300 MHz, DMSO-d$_6$, 25 °C, TMS): δ = 1.21 (s, 72H, 24×CH_3), 3.72+3.63 (br 2s, 40H, 20×CH_2), 7.40 (s, 8H, 8×Ar-H), 8.09 (s, 4H, 4×Ar-H), 9.29 ppm (s, 8H, 8×NH); ^{13}C-NMR (300 MHz, DMSO-d$_6$, 25 °C, TMS): δ = 27.14, 42.68, 113.88, 135.73, 139.43, 164.73, 168.99, 176.61 ppm; MS: M/Z: calcd for $C_{94}H_{131}N_{24}O_{12}$ (M^+): 1790.2; found: 1790.9; elemental analysis: calcd (%) for ($C_{94}H_{132}N_{24}O_{12}$+7$H_2O$) C 58.92, H 7.68, N 17.54; found: C 58.72, H 7.51, N 17.42.

(t-Bu-G$_2$N)$_2$ was prepared in 69.3% . ^{1}H-NMR (300 MHz, DMSO-d$_6$, 25 °C, TMS): δ = 1.21 (s, 144H, 48×CH$_3$), 3.75+3.42 (br 2s, 104H, 52×CH$_2$), 7.42 (s, 16H, 16×Ar-H), 8.09 (s, 8H, 8×Ar-H), 9.30 ppm (s, 16H, 16×NH); ^{13}C-NMR (300 MHz, DMSO-d$_6$, 25 °C, TMS): δ = 27.14, 41.86, 42.68, 47.21, 113.55, 113.93, 135.71, 139.43, 164.77, 169.03, 176.61 ppm; MS: M/Z: calcd for $C_{206}H_{287}N_{60}O_{24}Na$ (M+Na-H)$^+$: 4010.9; found: 4009.9; elemental analysis: calcd (%) for ($C_{206}H_{288}N_{60}O_{24}$+10$H_2O$) C 59.35, H 7.45, N 20.16; found: C 59.45, H 7.50, N 20.18.

CC(t-Bu-G$_1$N)$_3$ was prepared in 63.8%. ^{1}H-NMR (300 MHz, DMSO-d$_6$, 25 °C, TMS): δ = 1.22 (s, 108H, 36×CH$_3$), 3.75+3.40 (br 2s, 72H, 36×CH$_2$), 7.42 (s, 12H, 12×Ar-H), 8.10 (s, 6H, 6×Ar-H), 9.36 ppm (s, 12H, 12×Ar-H); ^{13}C-NMR (300 MHz, DMSO-d$_6$, 25 °C, TMS): δ = 27.45, 41.88, 43.00, 47.47, 113.89, 114.24, 136.03, 139.75, 165.09, 169.33, 176.92 ppm; MS: M/Z: calcd for $C_{150}H_{209}N_{42}O_{18}Na$ (M+Na-H)$^+$: 2911.5; found: 2911.3; elemental analysis: calcd (%) for ($C_{150}H_{210}N_{42}O_{18}$+8$H_2O$) C 59.39, H 7.51, N 19.39; found: C 59.28, H 7.63, N 19.20.

S2. Preparation of dendrimer 2

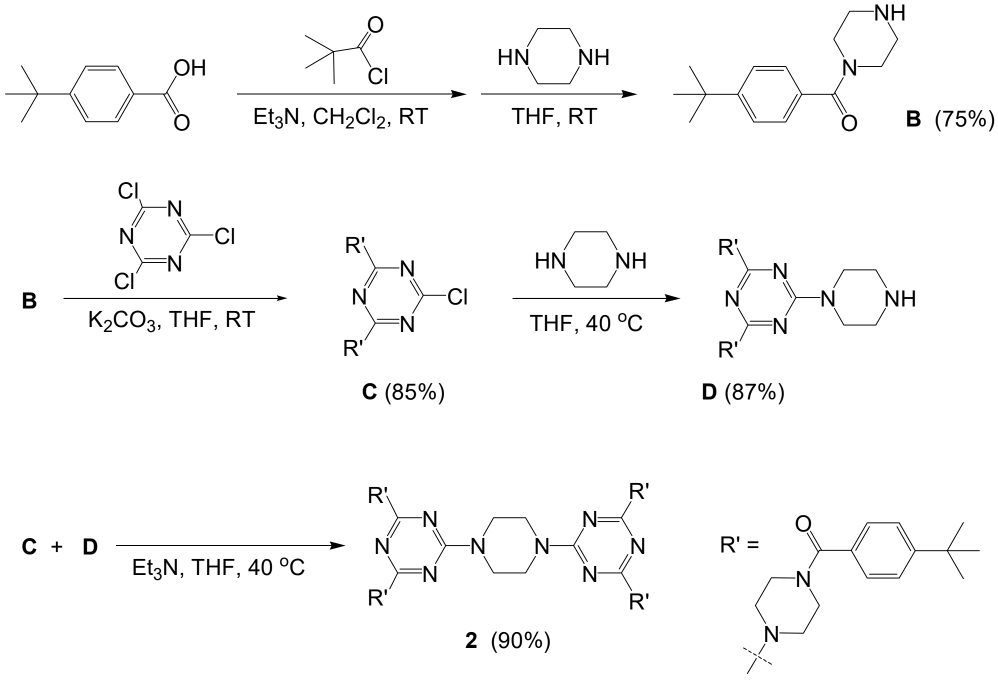

Figure S2. Preparation of dendrimer **2**.

Compound **B** was obtained in 75% yield in a similar manner to that of compound **A**, and compound **2** was synthesized from reaction of compound **C** and compound **D** accordingly in 90% yield (Figure S2). ^{1}H-NMR (300 MHz, $CDCl_3$, 25 °C, TMS): δ = 1.33 (s, 36H, 12×CH_3), 3.49 (br s, 8H, 4×CH_2), 3.76 (br s, 32H, 16×CH_2), 7.36 (d, 4H, *J*=8.4, 4×Ar-H), 7.43 ppm (d, 4H, *J*=8.4, 4×Ar-H); ^{13}C-NMR (300 MHz, $CDCl_3$, 25 °C, TMS): δ = 31.20, 34.83, 42.23, 43.08, 125.45, 126.95, 132.64, 153.18, 165.36, 170.82 ppm; MS: M/Z: calcd for $C_{70}H_{93}N_{16}O_4$ $(M+H)^+$: 1222.6; found: 1223.3; elemental analysis: calcd (%) for ($C_{70}H_{92}N_{16}O_4$) C 68.82, H 7.59, N 18.35; found: C 68.69, H 7.74, N 18.15.

S3. TGA analysis of dendrimers

(a)

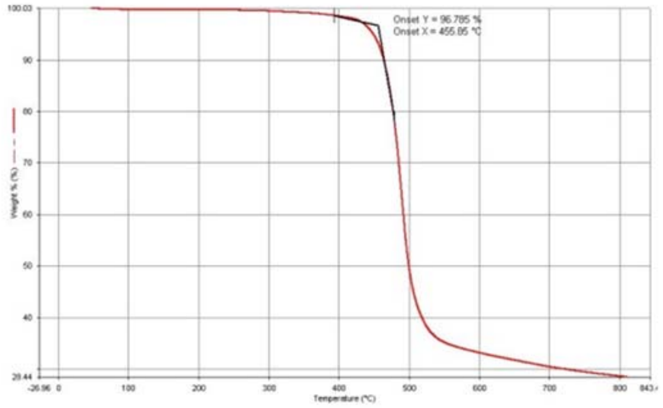

(b)

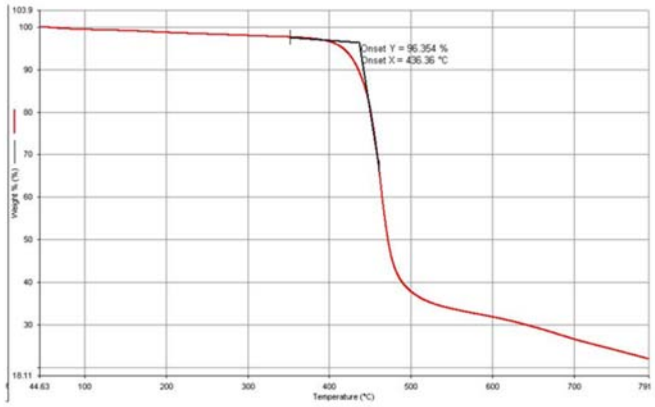

(c)

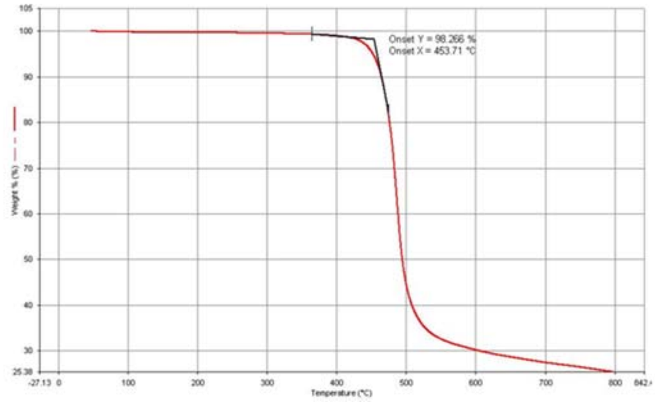

Figure S3. TGA analysis of (a) **(*t*-Bu-G$_2$N)$_2$**, (b) **CC(*t*-Bu-G$_1$N)$_3$**, and (c) **(*t*-Bu-G$_1$N)$_2$** at the heating rate of 2 °C min^{-1} under N_2 (sample was preheated to remove moisture).

S4. TGA analysis of 2

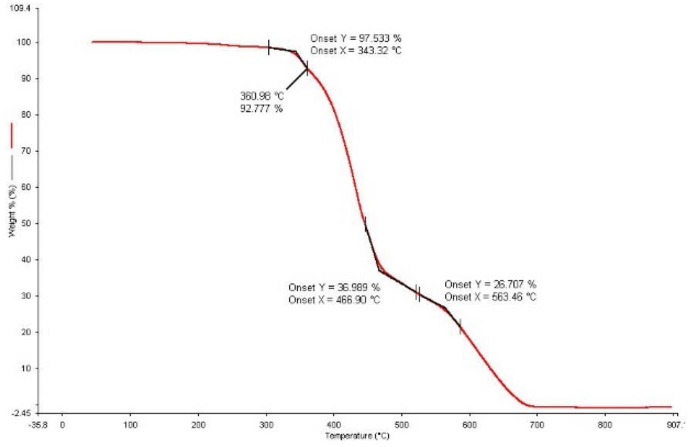

Figure S4. TGA analysis of **2** at the heating rate of 10 °C min^{-1} under N_2 (sample was preheated to remove moisture).

S5. Estimation of isosteric heats of gas sorption

A virial-type expression comprising the temperature independent parameters a_i and b_i was employed to calculate the enthalpies of sorption for CO_2 (at 273 and 298 K) on **(*t*-Bu-G$_2$N)$_2$, CC(*t*-Bu-G$_1$N)$_3$** and **(*t*-Bu-G$_1$N)$_2$**. The data were fitted using the equation:

$$\ln P = \ln N + 1/T \sum_{i=0}^{m} a_i N^i + \sum_{i=0}^{n} b_i N^i \qquad (1)$$

Here, P is the pressure, N is the amount adsorbed (or uptake), T is the temperature, a_i and b_i are virial coefficients, and m, n represent the number of coefficients required to adequately describe the isotherms (m and n were gradually increased until the contribution of extra added a and b coefficients was deemed to be statistically insignificant towards the overall fit, and the average value of the squared deviations from the experimental values was minimized). The values of the virial coefficients a_0 through a_m were then used to calculate the enthalpy heats of adsorption using the following expression.

$$Q_{st} = -R \sum_{i=0}^{m} a_i N^i \qquad (2)$$

Q_{st} is the coverage-dependent isosteric heat of sorption and R is the universal gas constant. The heat of CO_2 sorption for **(*t*-Bu-G$_2$N)$_2$**, **CC(*t*-Bu-G$_1$N)$_3$** and **(*t*-Bu-G$_1$N)$_2$** in this manuscript is determined by using the excess sorption data in the pressure range from 0−1 atm (273 and 298 K), which is fitted by the virial-equation very well ($R^2 > 0.9999$, Figure S6).

S6. The CO_2 isotherms for dendrimers

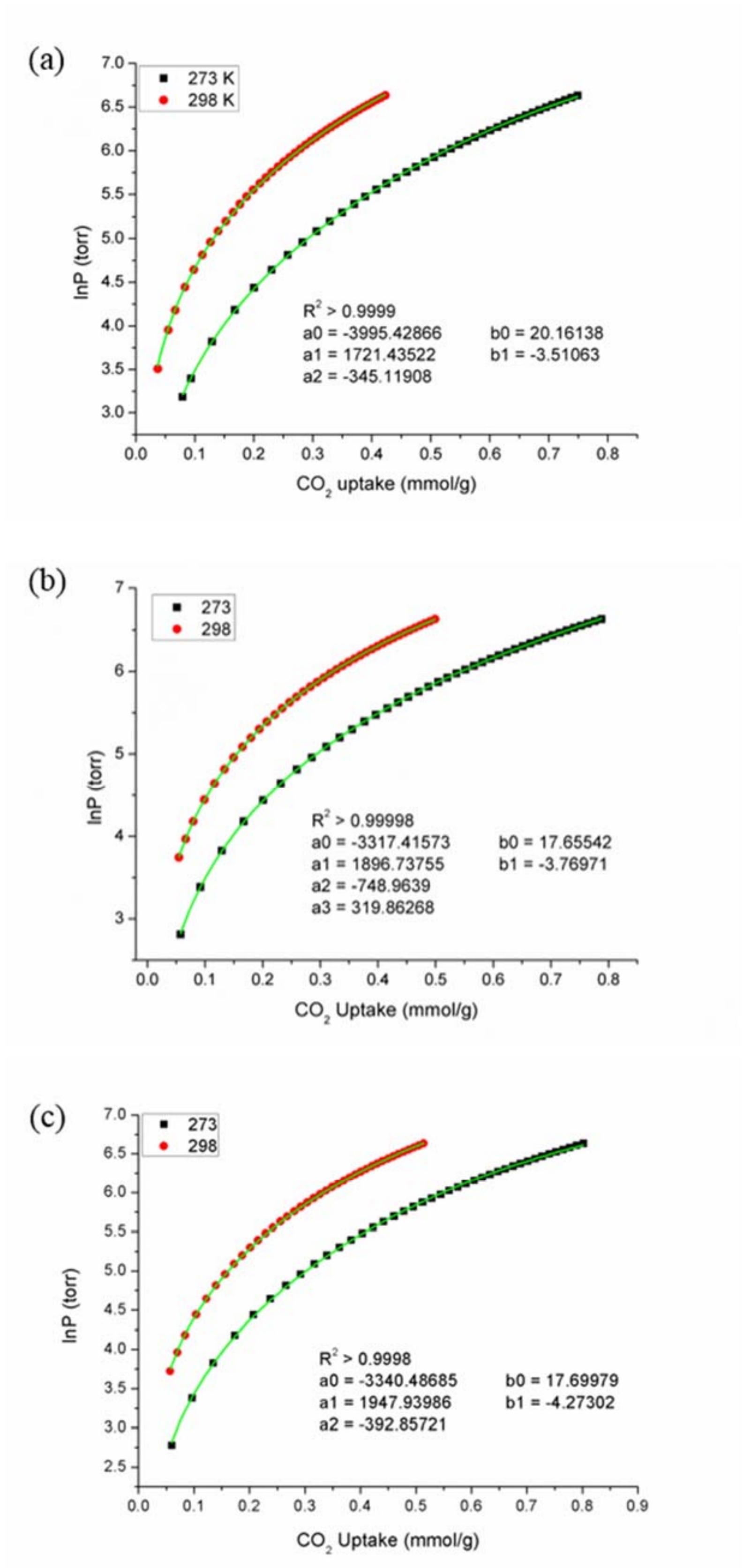

Figure S6. The CO_2 isotherms of (a) **(*t*-Bu-G$_2$N)$_2$**, (b) **CC(*t*-Bu-G$_1$N)$_3$** and (c) **(*t*-Bu-G$_1$N)$_2$** at 273 K (black) and 293 K (red) and the virial equation fits (lines).

S7. FT-IR and powder-XRD data for 2

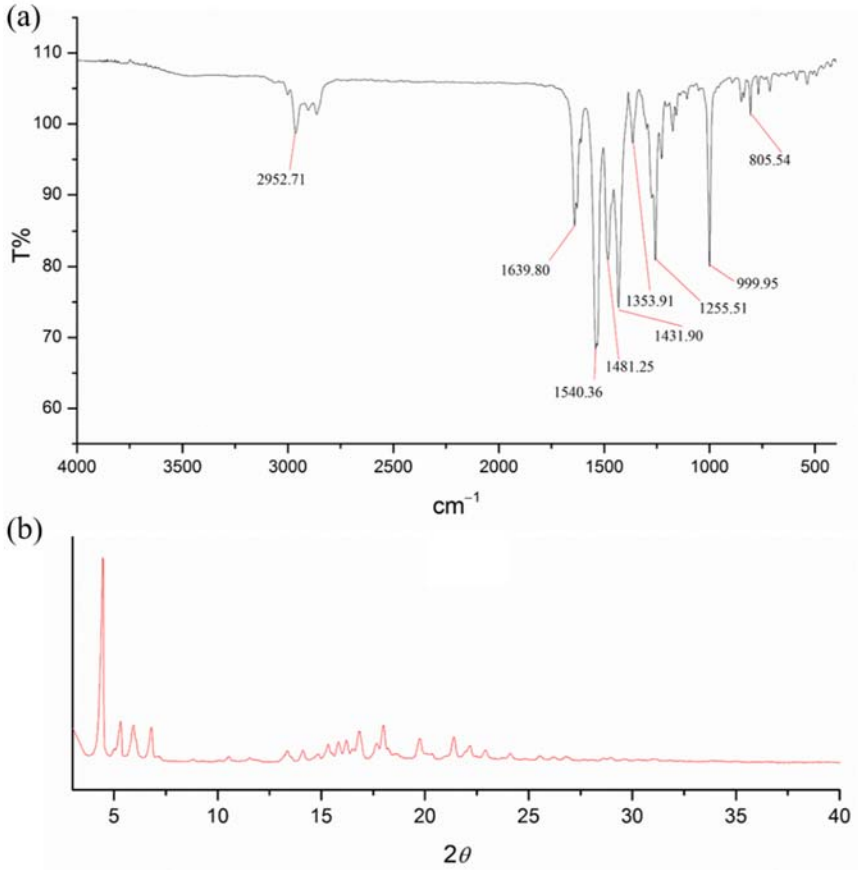

Figure S7. The FT-IR spectrum (a) and powder-XRD pattern (b) of compound **2**.

S8. CO_2 isotherms of 2

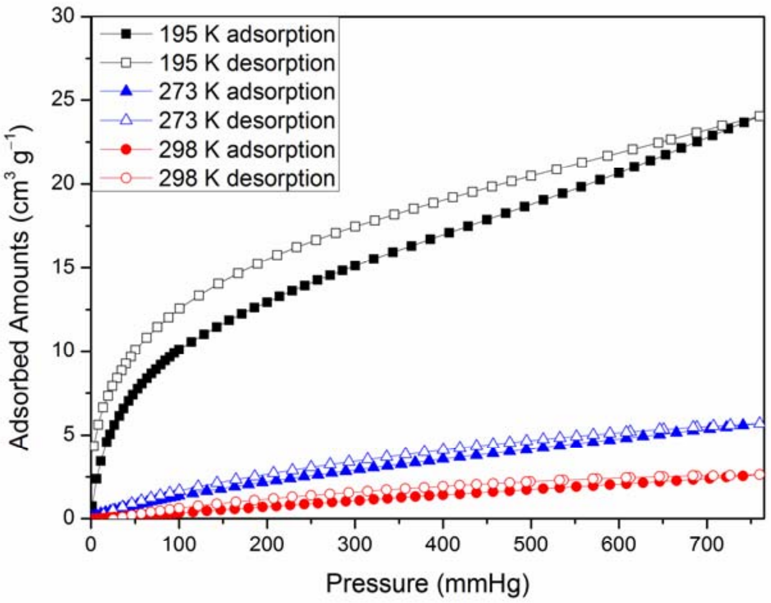

Figure S8. The CO_2 isotherms of **2** at 195 K, 273 K and 298 K.

S9. Crystal structure of 1

Table S1. Crystal Data and Structure Refinement Results for **1**

	**1**
chemical formula	$C_{26}H_{40}N_4O_4$
formula weight	472.62
crystal system	Monoclinic
space group	$P2_1/c$
temperature	155
a (Å)	10.6219(5)
b (Å)	28.3067(14)
c (Å)	9.2586(4)
β (°)	108.9310(10)
V (Å^3)	2633.2(2)
Z	4
D_{calcd} (g /cm^3)	1.192
θ range/(°)	2.96 - 26.42
μ (mm^{-1})	0.081
F (000)	1024
reflns collected	40517
unique reflns	5380
parameters	315
R_{int}	0.0492
R_1, wR_2[a] ($I > 2\sigma(I)$)	0.0514, 0.1336
R_1, wR_2[a] (all data)	0.0687, 0.1498
GOF	1.075

[a] $R1=\Sigma||F_0|-|F_c||/\Sigma|F_0|$; $wR2=[\Sigma w(F_0^2-F_c^2)^2/\Sigma w(F_0^2)^2]^{1/2}$

S10 NMR Spectra

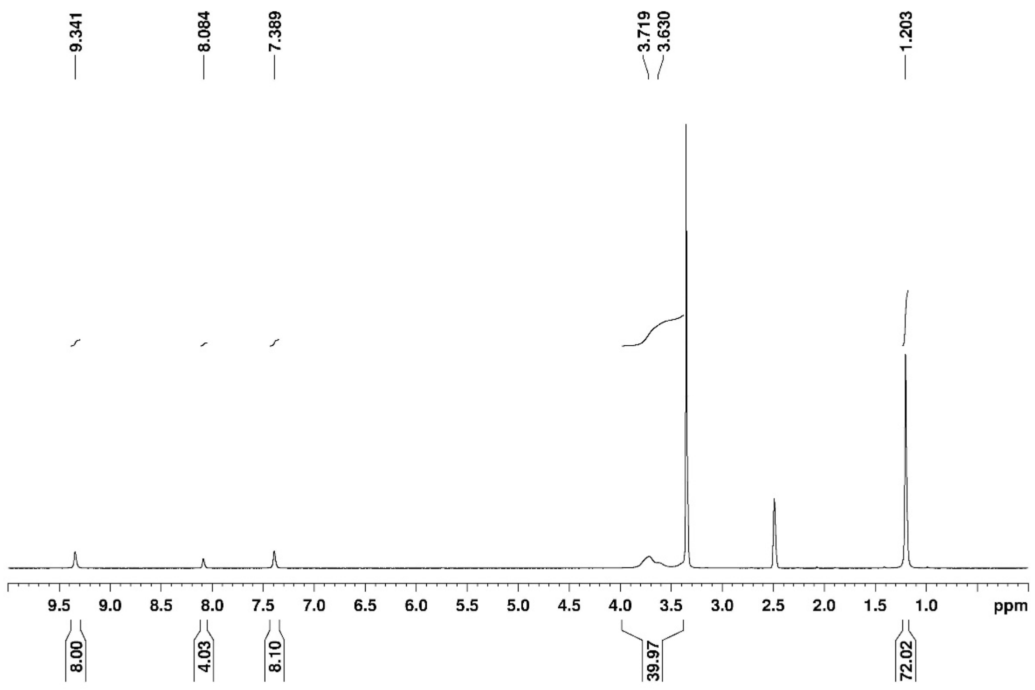

^{1}H-NMR spectrum of **(*t*-Bu-G$_1$N)$_2$**.

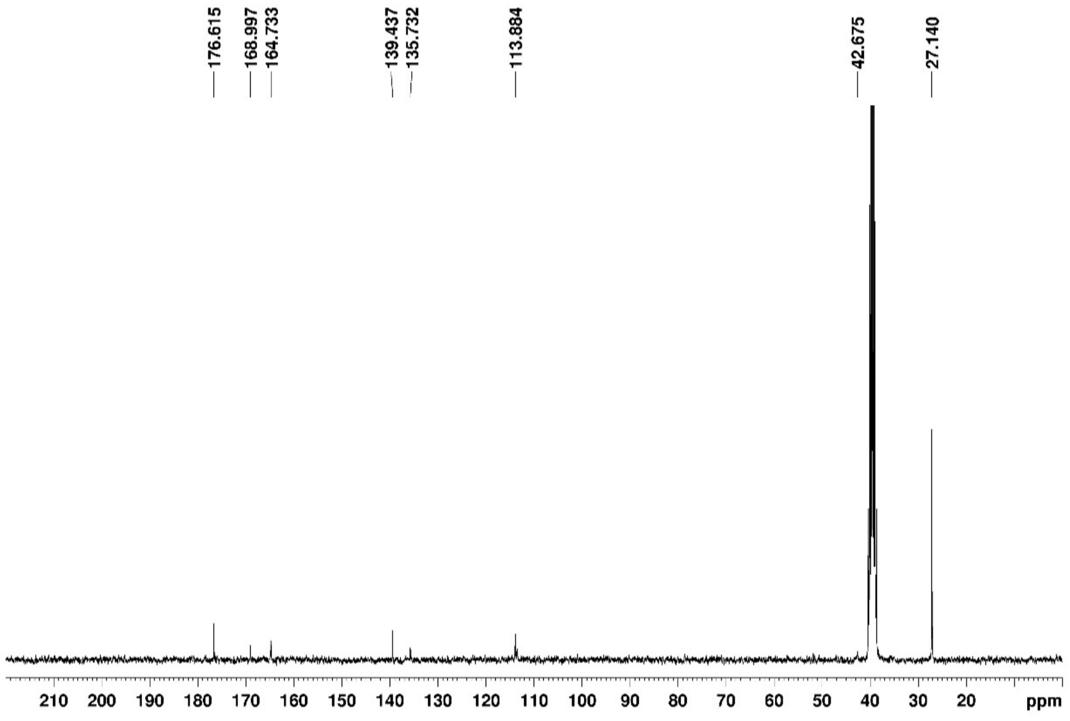

^{13}C-NMR spectrum of **(*t*-Bu-G$_1$N)$_2$**.

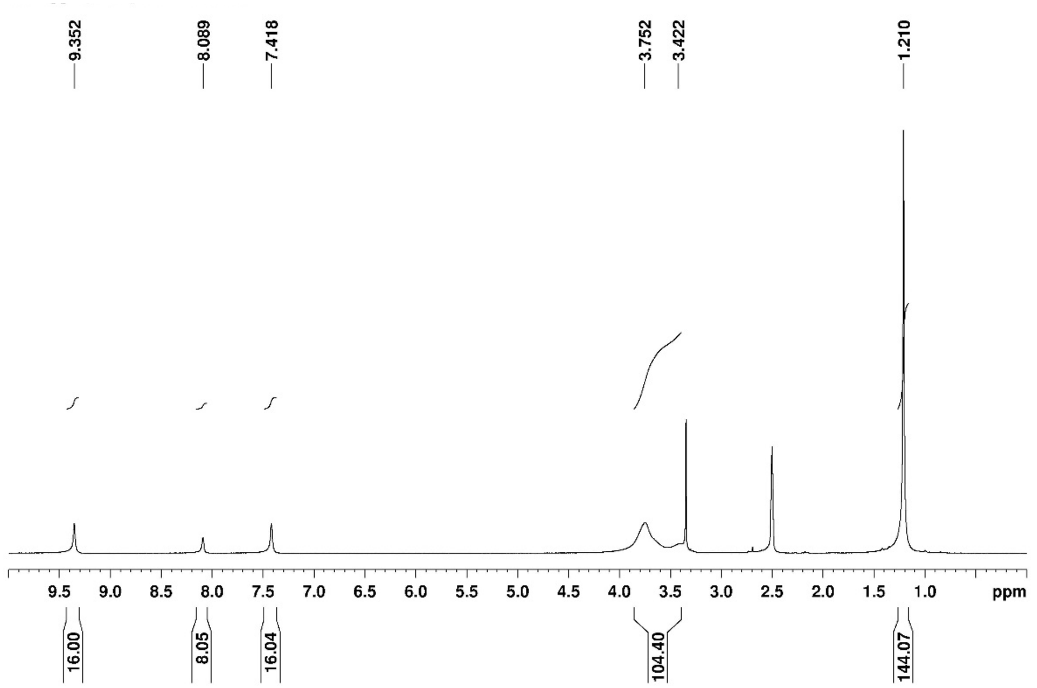

^{1}H-NMR spectrum of **(*t*-Bu-G$_2$N)$_2$**.

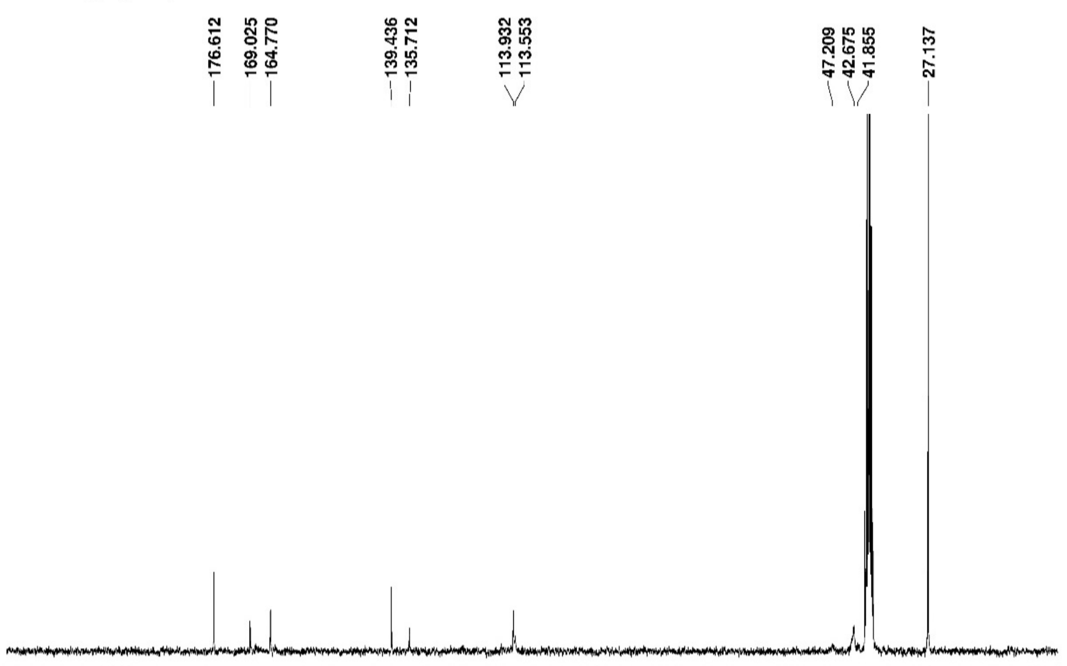

^{13}C-NMR spectrum of **(*t*-Bu-G$_2$N)$_2$**.

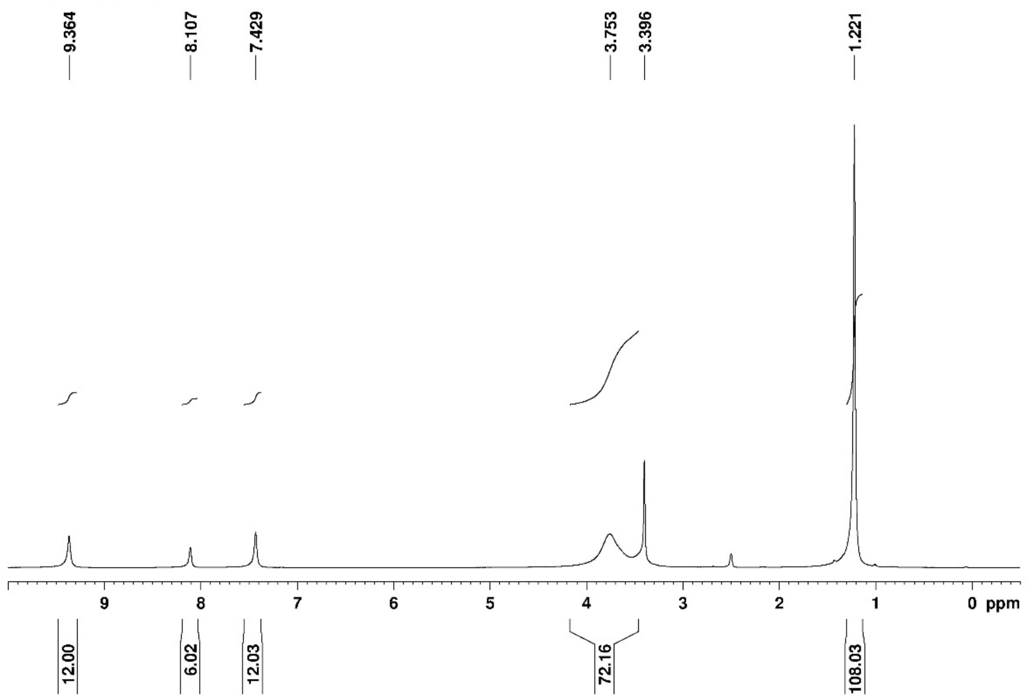

^{1}H-NMR spectrum of **CC(*t*-Bu-G$_1$N)$_3$**.

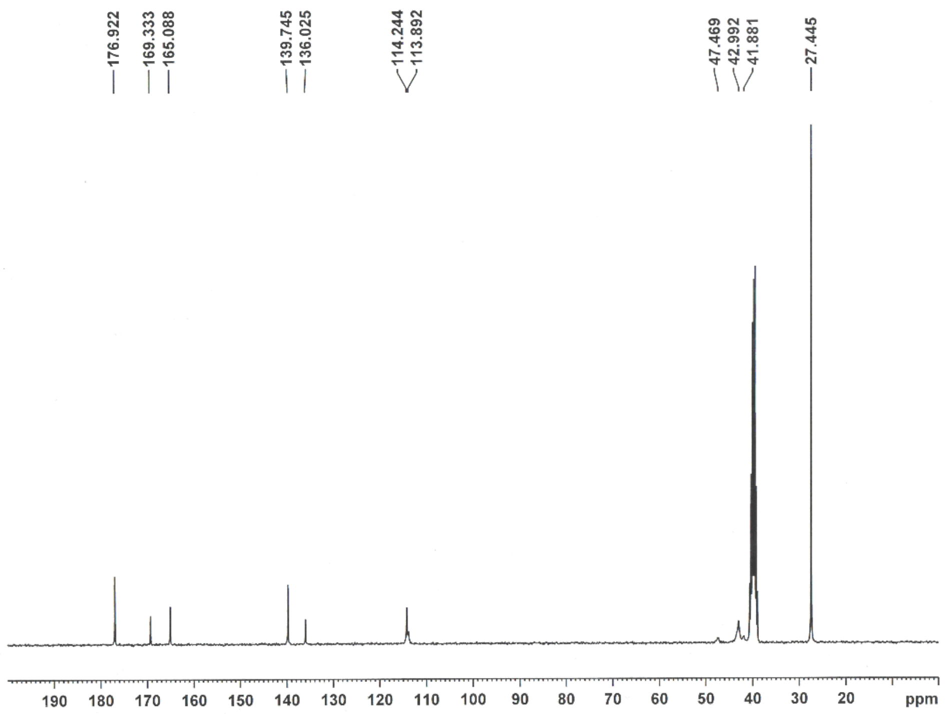

^{13}C-NMR spectrum of **CC(*t*-Bu-G$_1$N)$_3$**.

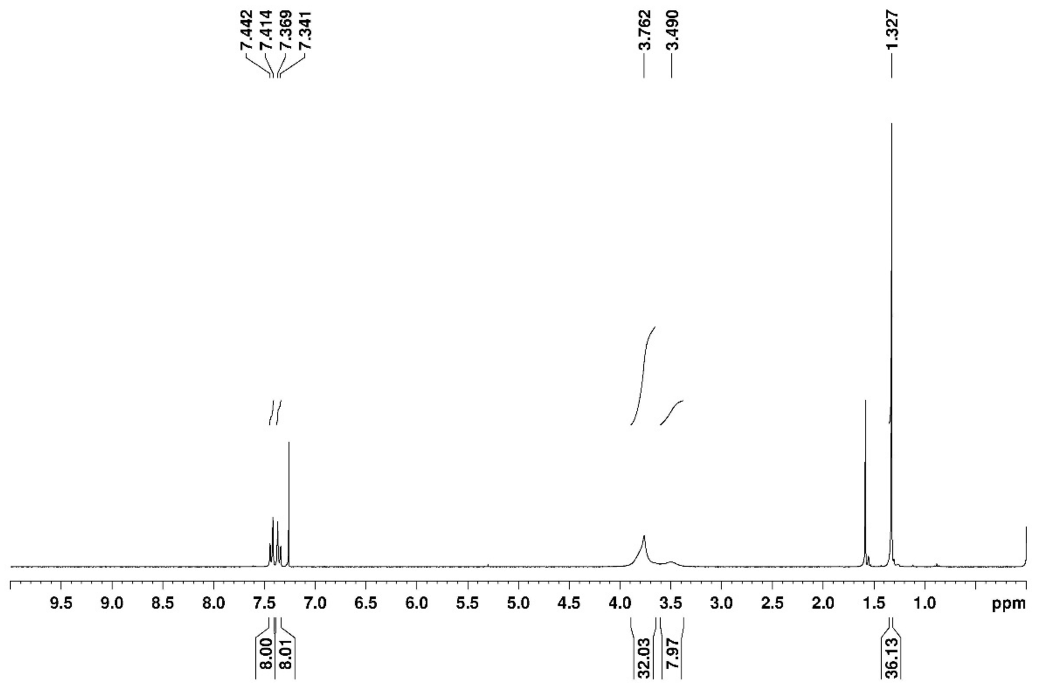

^{1}H-NMR spectrum of **2**.

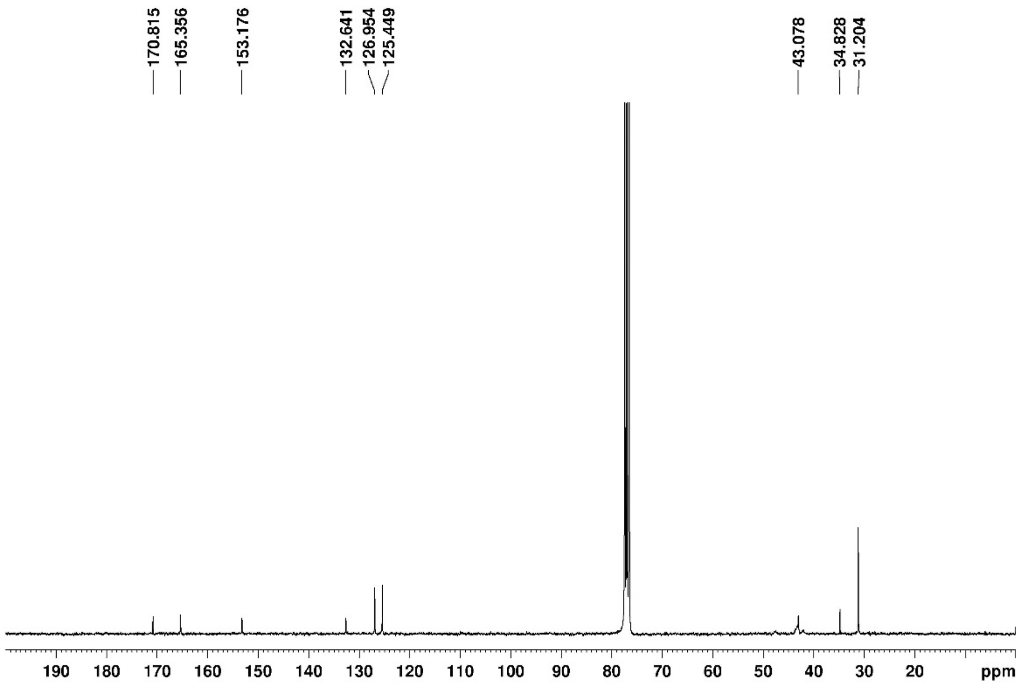

^{13}C-NMR spectrum of **2**.
